# Functional and Structural Divergence of an Unusual LTR Retrotransposon Family in Plants

**DOI:** 10.1371/journal.pone.0048595

**Published:** 2012-10-31

**Authors:** Dongying Gao, Jose C. Jimenez-Lopez, Aiko Iwata, Navdeep Gill, Scott A. Jackson

**Affiliations:** 1 Center for Applied Genetic Technologies, University of Georgia, Athens, Georgia, United States of America; 2 Department of Biochemistry, Cell & Molecular Biology of Plants, Estacion Experimental del Zaidin, High Council for Scientific Research, Granada, Spain; 3 Department of Botany, University of British Columbia, Vancouver, British Columbia, Canada; University of Arizona, United States of America

## Abstract

Retrotransposons with long terminal repeats (LTRs) more than 3 kb are not frequent in most eukaryotic genomes. Rice LTR retrotransposon, *Retrosat2*, has LTRs greater than 3.2 kb and two open reading frames (ORF): ORF1 encodes enzymes for retrotransposition whereas no function can be assigned to ORF0 as it is not found in any other organism. A variety of experimental and *in silico* approaches were used to determine the origin of *Retrosat2* and putative function of ORF0. Our data show that not only is *Retrosat2* highly abundant in the *Oryza* genus, it may yet be active in rice. Homologs of *Retrosat2* were identified in maize, sorghum, Arabidopsis and other plant genomes suggesting that the *Retrosat2* family is of ancient origin. Several putatively cis-acting elements, some multicopy, that regulate retrotransposon replication or responsiveness to environmental factors were found in the LTRs of *Retrosat2*. Unlike the ORF1, the ORF0 sequences from *Retrosat2* and homologs are divergent at the sequence level, 3D-structures and predicted biological functions. In contrast to other retrotransposon families, *Retrosat2* and its homologs are dispersed throughout genomes and not concentrated in the specific chromosomal regions, such as centromeres. The genomic distribution of *Retrosat2* homologs varies across species which likely reflects the differing evolutionary trajectories of this retrotransposon family across diverse species.

## Introduction

Long terminal repeat (LTR) retrotransposons are the most prominent mobile sequence in many plants. They replicate via an RNA intermediate and can rapidly increase in copy number resulting in large differences in genome sizes between related species (C-value enigma) [1]. For instance, the genome size of maize (*Zea mays*, 2n = 2x = 20) is six times larger than rice (*Oryza sativa*, 2n = 2x = 24) due primarily to massive amplifications of LTR retrotransposons in maize after the split of the two species [2], [3]. LTR retrotransposons contain direct LTRs that flank the internal sequences and are similar to retroviruses in structure and replication cycle, although they are unable to move from cell to cell [4]. The internal regions of retrotransposons encode *gag* and *pol* polyproteins that catalyze transpositon. Based on sequence divergence and the order of encoded gene products, LTR retrotransposons have been subdivided into *Ty1-copia* and *Ty3-gypsy* superfamilies [5]. Some retrotransposons from both *Ty1-copia* and *Ty3-gypsy* superfamilies have an additional open reading frame (ORF) located between the ORF encoded *gag-pol* polyproteins and 3′LTR, which encodes a retroviral envelope-like protein (*env*) [6], [7]. However, the function of the *env* gene in plants is not clear.

Unlike the internal regions, the LTRs do not code for enzymes for the replication of retrotransposons they do, however, play a critical role in controlling the activity of LTR retrotransposons. LTRs are composed of three distinct regions (U3, R and U5) that harbor regulatory factors including enhancers, promoters, and termination signals [8], [9]. For instance, a 10-bp sequence divergence in U3 region can result in significant differences in promoter activity [10]. In addition, some LTRs harbor cis-regulatory signals that confer responsiveness to various external stimuli and play a role in reactivation of transposition [11]. Moreover, LTRs from several retrotransposons in yeast have been found to contain sequences complementary to the primer binding sequence (PBS) allowing self primed reverse transcription [12]. Intriguingly, LTRs can serve as alternative promoters or enhancers to regulate genes as far away as 40–70 kb [13]–[15]. Despite their functional conservation, LTRs evolve rapidly and are highly divergent. In addition, the LTR sizes are extremely variable and can range from 80 bp [16], [17] to more than 5 kb [18]. The evolutionary origins and biological functions of larger LTRs are poorly understood.

Massive proliferation of LTR retrotransposons not only results in genome expansion but can also result in deleterious or lethal mutations. Thus, several mechanisms have evolved to suppress retrotransposon activity. This includes an epigenetic silencing mechanism by which transposons are either methylated thereby suppressing transcriptional activity or targeting of transposon transcripts by small RNAs (sRNAs) for post-transcriptional silencing [19]. Retrotransposons may, however, have insertion biases for genomic locations neutral to the host organism which allow the retrotransposon to persist [20]. For example, the *Ty3* LTR retrotransposon in *Saccharomyces cerevisiae* usually targets regions upstream of RNA polymerase III genes and may be harmless for the genome [20], [21]. In rice, the low copy but still active copia-like retrotransposon, *Tos17*, appears to prefer to insert (or be retained) into genic regions [22]. However, many high copy retrotransposons are concentrated in heterochromatic regions (centromeres, pericentromeres, telomeres) [23]. The distribution patterns of transposon families can be conserved across related genomes. A prominent example is the centromeric retrotransposons (CRs) family in grass species that is highly conserved over long evolutionary periods among a majority of the grass species and concentrated in centromeric regions of rice, wild rice, wheat, barley and maize [24]–[28].

We recently demonstrated that canonical CRs were absent in the wild rice species, *O. brachyantha*, and replaced by a novel retrotransposon, *FRetro3* [29]. *FRetro3* has no sequence similarity with the CRs in the grass genomes it does, however, has sequence similarity with a retroelement in rice, *Retrosat2*. Both *FRetro3* and *Retrosat2* are large elements (more than 12 kb) and have LTRs longer than 3.2 kb. Intriguingly, they both contain an extra ORF named ORF0 located upstream of the ORF encoding the *gag*-*pol* proteins (ORF1). The ORF0 sequence appears to be unique in rice as no other LTR retrotransposon from any organism contains a similar ORF0. The origin and function of the ORF0 sequence is not known.

We used a combination of comparative genomics, protein structure predictions and experimental approaches to gain insight into the function and evolution of this unusual family of retrotransposons, *Retrosat2*. Our results indicate that the *Retrosat2* family is abundant in the *Oryza* genus and may still be active. Homologs of Retrosat2 were found in maize, sorghum, Arabidopsis and other plant genomes suggesting an ancient evolutionary origin. In contrast to *FRetro3*, *Retrosat2* and its homologous elements were dispersed throughout the host genomes. Sequence analysis and 3D protein structural study of ORF0 sequences from *Retrosat2* and other homologs reveal that the ORF0 sequences have evolved more quickly than the retrotransposases. Finally, we show that the genomic distribution and evolutionary functions of this transposon family have likely experienced divergent selection pressures among these various plant genomes.

## Results

### *Retrosat2* is abundant and dispersed throughout the rice genome

**Figure 1:**
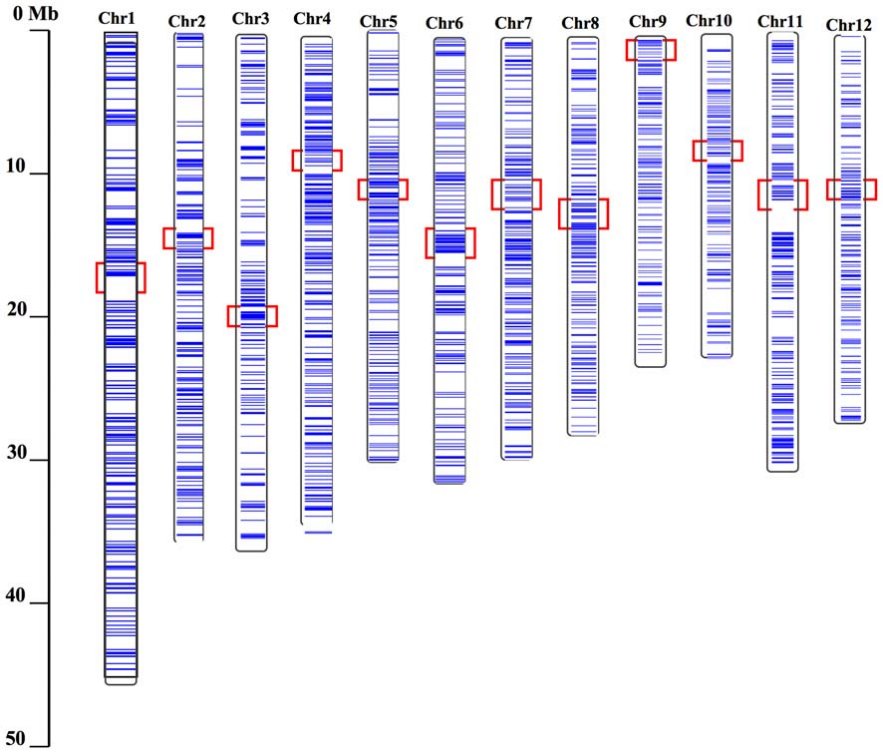
Distributions of *Retrosat2* elements on 12 chromosomes of Nipponbare. Blue horizontal lines indicate Retrosat2 elements. Red rectangles mark the centromeric positions of 12 rice chromosomes.

*Retrosat2* was originally identified as a nested LTR retrotransposon (Accession number: AF111709) with little other genomic description. We annotated the Nipponbare (*Oryza sativa* L. ssp. *japonica*) genome and identified more than 1000 complete or partial copies of *Retrosat2*, including 162 complete elements and 429 solo-LTRs. Together, these elements comprise about 1.4% of the Nipponbare genome (Table S1). The genome-wide ratio of solo-LTR to complete element is about 2.6∶1 (429/162), although the ratio varies among the 12 chromosomes. Chromosome 3 has the highest ratio of solo-LTR to complete element (42/4 = 10.5∶1), whereas, chromosome 11 has the lowest (29/18 = 1.6∶1). Further investigation indicated that Retrosat2 elements are dispersed unevenly throughout the rice genome (Figure 1).

**Figure 2:**
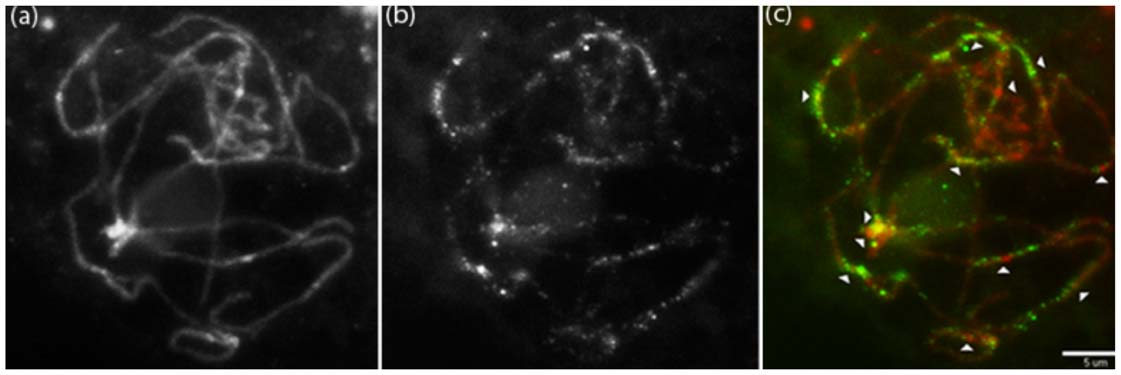
FISH image of 1.5 kb LTR sequence of *Retrosat2* on pachytene chromosomes. (a) 4′,6-diamidino-2-phenylindole (DAPI) counterstain, (b) *Retrosat2*, (c) Merged image. Chromosomes were psuedocolored red and Retrosat2 signals are shown green. Arrows indicate locations of 12 centromeres and the scale bar represents 5 µm.

To provide more insight into chromosomal distribution of *Retrosat2*, fluorescence *in situ* hybridization (FISH) using the LTR of *Retrosat2* as a probe was performed. Some FISH signals derived from Retrosat2 flanked or overlapped the centromeres (Figure 2), but the majority of the signals were dispersed on chromosomal arms which confirm that *Retrosat2* is not concentrated at specific chromosomal regions but rather is distributed across the genome. It is interesting that the chromosomal distribution of *Retrosat2* differs from its homolog, *FRetro3*, in *O. brachyantha* which is enriched in peri- and centromeric regions [29].

### *Retrosat2* elements are present throughout the *Oryza* genus

The *Retrosat2* sequence was used to query GenBank to determine if *Retrosat2*-like elements are present in other *Oryza* species. Complete *Retrosat2* homologs were found in *O. rufipogon* (*Sat2-ruf*), *O. punctata* (*Sat2-pun*), *O. minuta* (*Sat2-min*), *O. officinalis* (*Sat2-off*) and *O. australiensis* (*dingo*, [30]) and ranged in size from 11,375 to 13,324 bp (Table S2). No complete element was found in *O. glaberrima* or *O. granulata*; however, solo-LTR or fragments (>3 kb) were identified in both genomes with more than 80% sequence similarity to *Retrosat2*. These results suggest that *Retrosat2* and its homologs are present throughout the *Oryza* genus.

We use the BAC-end sequences (BESs) from 12 *Oryza* genomes generated by the Oryza Map Alignment Project (OMAP) (http://www.omap.org) to detect the distribution and abundance of *Retrosat2* family in the genus *Oryza*. This survey may not reflect the real situation of *Retrosat2* in the genus as BESs only cover the regions having a restriction site for the enzyme that was used to build the BAC libraries However, whole genome sequences are not available for all *Oryza* species, only available for *Oryza sativa*. Elements from the *Retrosat2* family were used to search against the BESs. *Retrosat2* elements were identified in all 12 genomes, but the abundance varies dramatically among the 12 genomes. For example, *Retrosat2* elements account for 3.72% of the *O. australiensis* BESs but only 0.03% of *O. coarctata* (Table S3).

**Figure 3:**
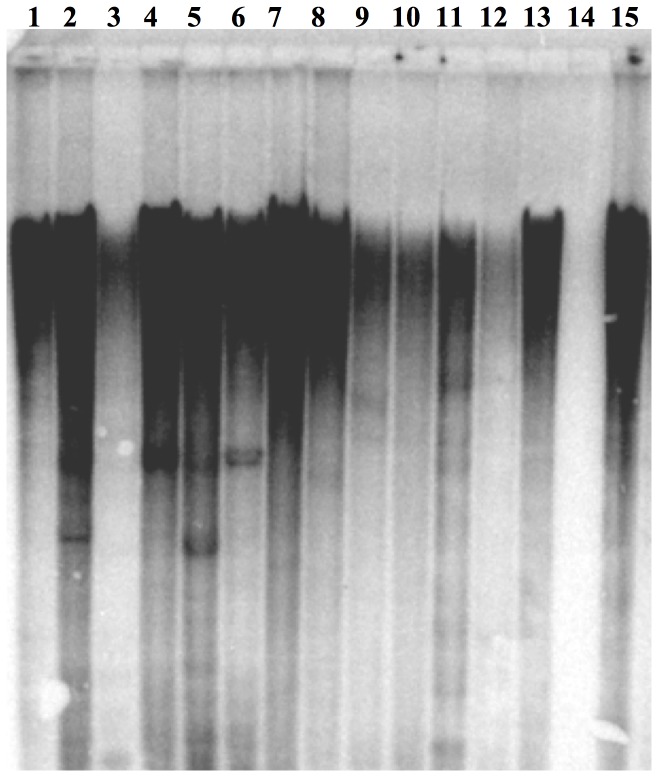
Southern blot of Retrosat2. (1) Oryza brachyantha, (2) Oryza sativa (Nipponbare), (3) Oryza glaberrima, (4) Oryza nivara, (5) Oryza longistaminata, (6) *Oryza rufipogon*, (7) *Oryza minuta*, (8) *Oryza officinalis*, (9) *Oryza punctata*, (10) *Oryza alta*, (11) *Oryza australiensis*, (12) *Oryza granulata*, (13) *Oryza ridleyi*, (14) *Oryza coarctata* and (15) *Oryza brachyantha*.

In order to confirm the distribution of *Retrosat2* family in the *Oryza* genus, Southern blot analysis was performed. Strong signals were detected in all tested genomes except *O. coarctata* (Figure 3) confirming our results from BES analysis. No hybridization signal was found in *O. coarctata*, suggesting that *Retrosat2* elements are either present in very few copies, highly diverged or in the process of extinction.

### Integration times of *Retrosat2* in rice

**Figure 4:**
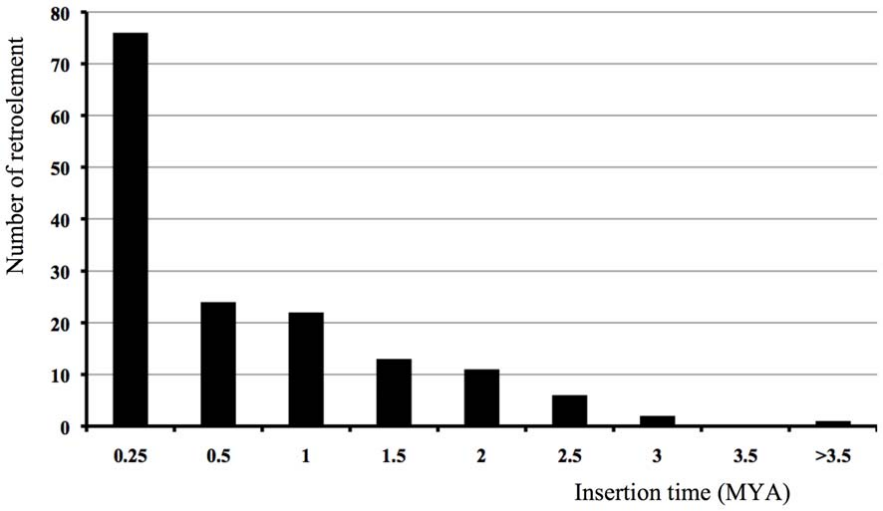
The insertion times of full-length *Retrosat2* elements in *O.* sativa cultivar Nipponbare.

Because LTR retrotransposons replicate via RNA intermediates and has a similar life cycle to the retrovirus, the LTRs should be identical at the time of insertion and then diverge independently through the accumulation of mutations. Thus, the sequence divergence of LTRs of a retrotransposon can be used to estimate the insertion time of the retroelement [31]. The insertion times of 155 complete LTR retrotransposons were calculated for the Nipponbare genome following published methodologies [32] and range from 0 to 3.73 million year ago (MYA). Most elements integrated in the last 0.5 MY, 76 and 24 elements within 0.25 and 0.5 MYA, respectively (Figure 4). This suggests that the majority of *Restrosat2* elements were recently inserted into the rice genome and that the element may still be active.

The *O. sativa indica* and *japonica* subspecies diverged from a common ancestor ∼0.2–0.4 MYA [32]. Since the insertion times of 49% (76/155) of complete elements of *Retrosat2* were less than 0.25 million year (MY), many of the elements may have inserted into the rice genome after the split of two subspecies. To test this hypothesis, we compared the insertion patterns of *Retrosat2* between 93–11 (*indica*) and Nipponbare (*japonica*). To do this, the presence/absence of orthologous insertions were confirmed by using each element and ∼100bp flanking sequence from 93–11 to search against the Nipponbare genome and the reciprocal using Nipponbare sequences to search against the 93–11 genome. We found 95 and 40 new insertions in Nipponbare and 93–11, respectively. To detect the transcriptional activity of *Retrosat2*, we conducted RT-PCR analysis and found that *Retrosat2* was expressed in leaf, sheath and flower (Figure S1).

### Multiple putative regulatory signals are found in the LTRs of *Retrosat2*

**Figure 5:**
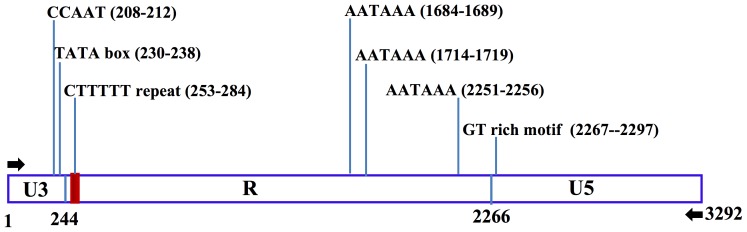
The sequence structure of *Retrosat2* LTR. The LTR is divided into three regions, U3 (1–244), R (245–2266) and U5 (2267–3292). Red rectangle denotes tandem repeats and black arrows indicate the 10-bp inverted terminal repeats of the LTR.

LTRs contain regulatory sequences and are important for the replication and integration of retrotransposons. The LTRs of *Retrosat2* are more than 3.2 kb and can be divided into the three canonical regions, U3, R and U5 (Figure 5). The *Retrosat2* U3 region contains the 5-bp enhancer motif (CCAAT) [8] found from nucleotides 208–212 and, in an 18-bp region after the enhancer signal, a TATA box for initiation of transcription. The R domain of *Retrosat2* is 2023 bp and starts with a G (nucleotide 244) and ends with CA (nucleotides 2064-65). The *Retrosat2* R region has unusual sequence features including: 1) Three polyadenylation (poly(A)) sites (AATAAA), located in nucleotides 1684–1689, 1714–1719 and 2251–2256; and 2) Seven tandem repeats of a CTTTTT motif. The U5 region contains GT or T-rich sequences usually located the first 40-bp region of the U5 domain that are thought to be important for retrotransposition [33], [34]. The G/T content of the first 31-bp region of the R domain is 90%, much higher than the average content (51.4%) of the rest of the *Retrosat2* LTR. In contrast to the U3 and R regions, the U5 region has sequence conservation between Retrosat2 and homologs within the *Oryza* genus (Figure S2).

Previous studies have shown that LTRs of the tobacco retrotransposon *Tto1* harbor signal sequences for responsiveness to various stresses [11]. Since the LTRs of *Retrosat2* are much larger than that of *Tto1* (574 bp), we suspected that the LTRs may contain regulatory sequences. To test this hypothesis, the LTRs of *Retrosat2* were used to search against PlantCARE (database of plant cis-acting regulatory elements [35]). Interestingly, the LTR sequence matched exactly 22 regulatory elements reported in rice and other plants (Table S4), including the C-repeat/DRE from Arabidopsis, Sp1 from rice and a TGACG-motif from barley. It should be noted that many of these elements are involved in stress responsiveness and that some cis-regulatory elements completely match multiple regions of the *Retrosat2* LTR. For instance, we found five CCGTCC sequences that matched the CCGTCC-box from Arabidopsis.

### Origin and phylogenetic analysis of the *Retrosat2* family

Complete elements or fragments of *Retrosat2* homologs were found across the *Oryza* genus (Tables S2 & S3) which indicate that the ancestor of *Retrosat2* likely existed before the divergence of the *Oryza* genus. To determine if *Retrosat2* homologs present outside the genus *Oryza*, the LTR, ORF0 and ORF1 of *Retrosat2* were used individually to search against GenBank and whole genome sequences from maize, sorghum, *Brachypodium distachyon* (Brachypodium), Arabidopsis, soybean, poplar, grape and *Selaginella moellendorffii*. No significant hits (E value <10^−5^) were found in these genomes using either the LTR or ORF0 as queries for BLAST searches. However, complete homologous elements, dispersed across the genomes, were identified in all eight genomes using the ORF1 sequence as a query. Interestingly, the homologs of Retrosat2 in maize (*ZMsat2*), sorghum (*Sorsat2*) and Brachypodium (*Brasat2*) also contain two ORFs, ORF0 and ORF1, whereas homologs in Arabidopsis (*Arasat2*), soybean (*Soysat2*), poplar (*Popsat2*) grape (*Grasat2*) and *Selaginella moellendorffii* (*Selsat2*) have only the ORF1. These results indicate that the ancestor of *Retrosat2* and its homologs predates the divergence of dicotyledonous and monocotyledonous plants.

The ORF1 sequences of *Retrosat2* and its homologs are more conserved than the ORF0 sequences. For example, the ORF1 proteins of *Retrosat2* and *Sorsat2* share more than 60% sequence similarity, whereas the ORF0 proteins from the two elements show ∼10% sequence similarity. To gain insight into the sequence divergence of ORF0 and ORF1 of *Retrosat2* and its homologs, we calculated the nonsynonymous (Ka) to synonymous (Ks) nucleotide substitution rates using the ORF0 and ORF1 sequences of *Retrosa2*, *Sa2-ruf*, *Sat2-min* and *Sat2-off*. *Dingo*, *FRetro3*, *ZMsat2*, *Sorsat2* and *Brasat2* were excluded as their ORF0 sequences were either too divergent or either ORF0 or ORF1 was truncated. The average Ka values of the ORF0 and ORF1 sequences are significantly different, p>0.005, at 0.2574 and 0.0783, respectively. The mean Ks values of the ORF0 and ORF1 sequences are 1.6471 and 1.3298, respectively, and not significantly different. Ka values usually reflect functional divergence whereas Ks values can be used to estimate divergence dates [36]. Thus, these results suggest the ORF0 and ORF1 likely diverged at the same time but have been subjected to different selection pressures.

**Figure 6:**
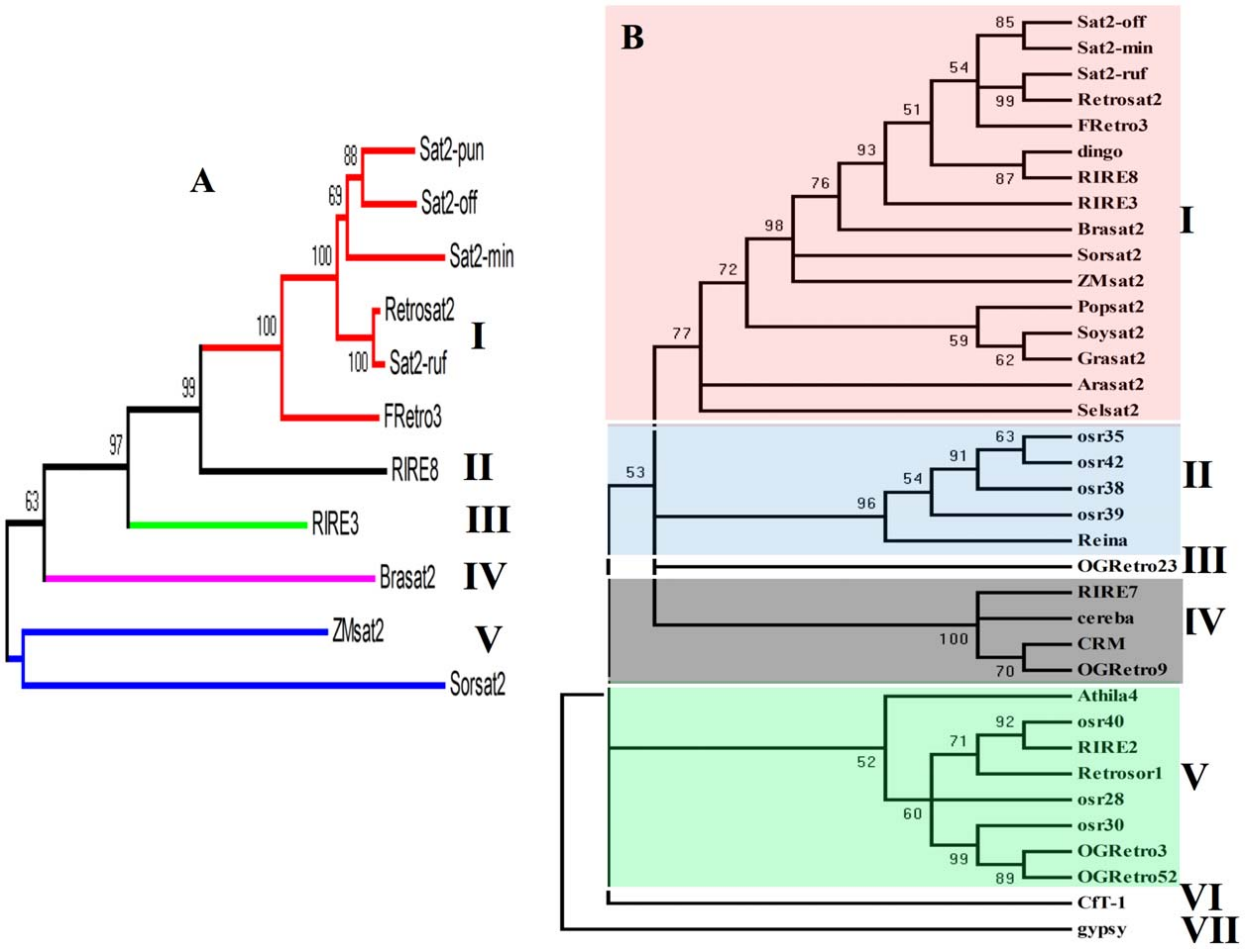
Phylogenetic trees based on the ORF0 sequences (A) and the RT conserved domains (B). Bootstrap values (>50%) are shown.

To illuminate the evolutionary relationship between *Retrosat2* and its homologs, two phylogenetic trees were constructed using the ORF0 proteins and the conserved reverse transcriptase (RT) domains of ORF1. The phylogenetic tree of ORF0 shows that the sequences fall into five subfamilies where *Retrosat2* and the *Oryza* genus homologs constitute a unique group (Figure 6A). The phylogenetic tree with the conserved RT domains indicates that these elements can be grouped into seven subfamilies. *Retrosat2* and all the homologs fall into the subfamily I, other plant retroelements were grouped into subfamilies II-V whereas *gypsy* from Drosophila and *CfT1* from *Passalora fulva* were grouped into subfamilies VI and VII, respectively (Figure 6B). Notably, *RIRE3* and *RIRE8* were grouped together with *Retrosat2* and its homologs suggesting that they likely diverged from a common ancestral family.

### Structural characterization and functional annotation of ORF0

**Figure 7:**
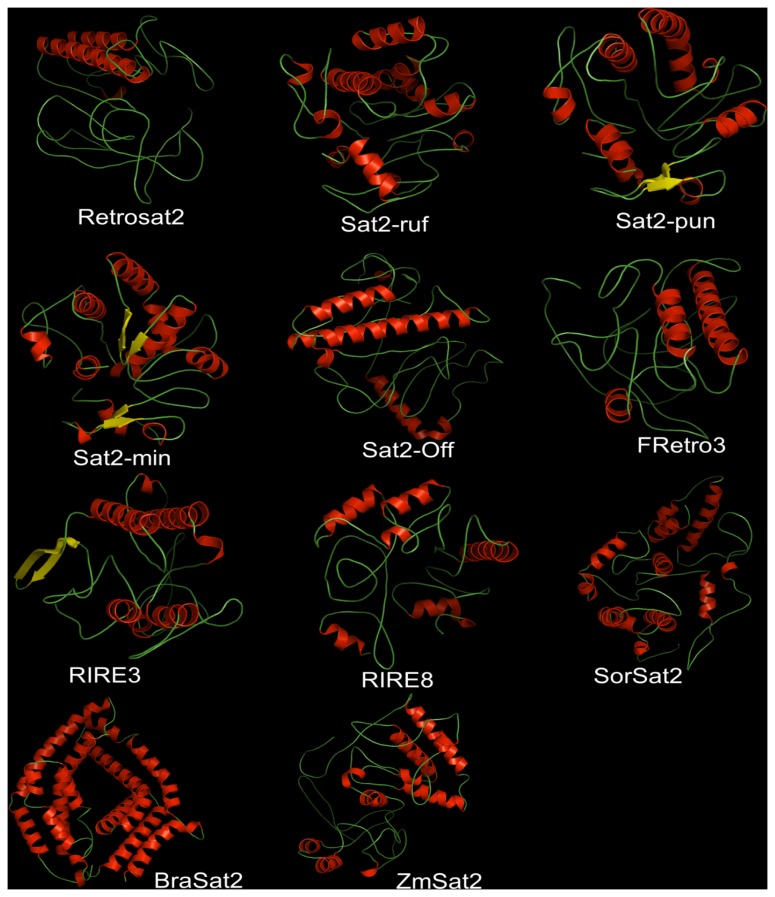
Three-dimensional structure analysis of the 11 ORF0 proteins. All structures are depicted as a cartoon diagram. Within the represented family, the secondary elements are colored in red (α-helix), yellow (β-sheet) and green (coils)

**Figure 8:**
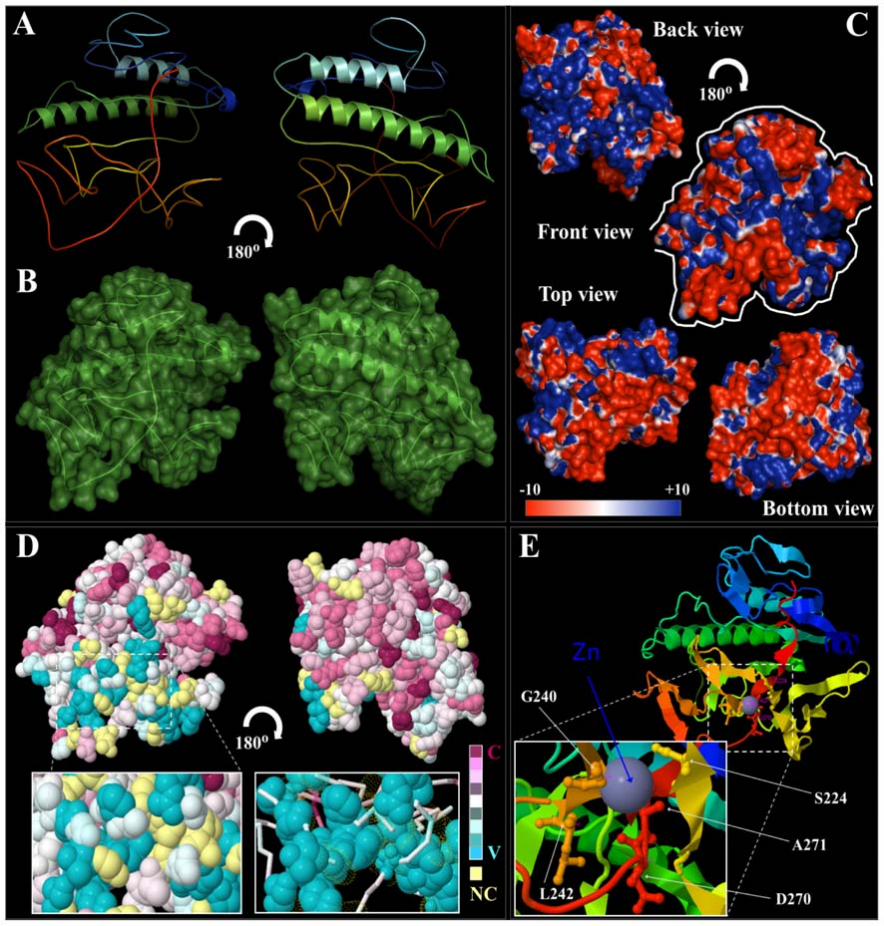
Detailed structural conformation and conservation analysis of *Retrosat2*, a rice ORF0 protein member. (A) General structure (cartoon diagram rainbow colored) shows the 2D structural elements of the rice *RetroSat2*, where N- and C-terminal are colored blue and red, respectively. Represented structures were rotated at 180u. (B) The surface conformation of *RetroSat2* (rotated 180u) showing the secondary structure elements inside is depicted. (C) Electrostatic surface potential showing front, back, top and bottom views of *RetroSat2* structure. The surface colors are clamped at red (−10) or blue (+10). Top and bottom views are highlighted with a white line coming from front view. (D) Best predicted *RetroSat2* model (2D-structure) was subject to consurf-conservational analysis searching for close homologous sequences with known structures using PSI-BLAST. The protein was finally visualized using FirstGlance in Jmol with the conservation scores being color-coded. The conserved and variable residues are presented as space-filled models and colored according to the conservation scores. A detailed view of the predicted ligand-binding cavity holding up the cofactor/ligand (van der Walls spheres and/or lines) is shown in high magnification. Represented structures were rotated at 180u. (E) Cartoon structural representation of a general front view of *RetroSat2* model (C- and N-terminal colored as blue and red respectively), showing the morphology of the predicted cofactor/ligand-binding pocket/cavity. A detailed view at higher magnification highlights the residues implicated in cavity formation and interaction with the ligand zinc (Zn), which are S224, G240, L242, D270, and A271.

Using computational modeling, we determined the structural features of the 3D general folds and compared the electrostatic surfaces, structural and key functional residues of different ORF0 proteins (Figures 7–8, Figures S3, S4, S5, S6). Because the native structures have not been crystallized, the structural similarity and accuracy of the models were further assessed using structural parameters: root mean square deviation (RMSD), sequence identity and E-value compared with the template, and Z-Score, (model reliability between 0–1). Their pseudo-energies of the contributing terms (C-beta interaction energy, all-atom pairwise energy, solvation energy and torsion angle energy), together with their Z-scores are the indicators of a well built model [37].

General structural comparisons (Figure 7) and phylogenetic analysis (Figure 6A) provide clear insights into the structural divergence of the ORF0 proteins. Following the minimum divergence criterion, RSMD comparisons between every pair of structures were measured (Table S5) indicating that deviations in structural comparisons analyzed were low for *Sat2-min*, *Sat2-pun*, *Sat2-ruf* and *Sat2-off* (Figure 7–8, Figures S4, S5, S6, Table S5).

Structural comparisons between the most closed structures (Figures 7, 8A, Figures S3A, S4, S5, S6A) are in line with the results obtained in the phylogenetic analyses (Figure 6A). Superimposition between the most similar members of the ORF0 family showed a close structural relationship. However, the special disposition for different 2D elements is the major structural divergence between *Sat2-min*, *Sat2-ruf*, *Sat2-pun* and *Sat2-off*. The greatest structural differences were found between *SorSat2* and *ZmSat2* (Table S5, Figure S3A, S4, S5, S6A). The Table S5 showed a strong structural correlation between the three structures *Sat2-min*, *Sat2-pun*, *Sat2-ruf*, as well as between *Retrosat2* and *Sat2-off*, based on the small RMSD values (3.99, 6.21, 6.60, and 5.693Å). Further comparisons between every pair of structures shown major similarities between *RIRE3* to *FRetro3* (RMSD = 8.508Å), *Sat2-min* to *BraSat2* (RMSD = 9.216 Å), *and RIRE8* to *SorSat2* (RMSD = 9.970Å).

Proteins folding and structural domains revealed that *SorSat2* and *ZmSat2* were the most divergent form of the current the ORF0 protein members (Figure 7, Table S5). Differences are also present in other many members of the ORF0 proteins when comparing the general surface domains, cavities and clefts modeled as consequence of the structural parameters variation, and spatial distribution of α-helices, β-sheets, coils, turns and other structural elements of the proteins (depicted in Figure 7, Figures 8B, Figures S3B, S4, S5, S6B).

The Adaptive Poisson-Boltzmann Solver (APBS) package [38] was used to generate the electrostatic surface potentials for all the ORF0 proteins (Figures 8C, Figures S3C, S4, S5, S6C). Although the overall topologies of these proteins are similar between some members of the ORF0 genes, such as *Sat2-min*, *Sat2-pun* and *Sat2-ruf*, several differences can still be observed. A specific electrostatic potential distribution pattern in the surface was observed for *Sat2-min* and *Sat2-pun*, as well as for *Retrosat2* and *Sat2-off*, with a abundance of negative and positive charge in the protein surfaceshowing numerous more differences ORF0 protein members, as were depicted in the general view (Figure 7), and isocontour representation data (Figures 8C, Figures S3C, S4, S5, S6C). The surfaces of the ligand-binding domains and the cofactor-binding domains contained the most profound differences in charge distributions inside the ORF0 protein members. However, charge distribution patterns (isocontour ranging from +10 kT to −10 kT) might correlate with differences and/or similarities in their activity, ligand-binding or protein-protein interaction [39], which also denotes differences in the mechanism of action and/or interaction with other proteins and intracellular targets [40].

The conservational analysis of residue implicated in the structural maintenance revealed similar residue patterns in all ORF0 protein members (Figures 8D, Figures S3D, S4, S5, S6D), where *Retrosat2* and *BraSat2* were slightly more conserved than the rest of the ORF0 members, with the most variable surface residues (depicted in blue) located on the periphery and the conserved residues (depicted in purple) located in the core of the protein structures. The most conserved residues were confined to the catalytic cleft of some ORF0 proteins structures. The most conserved environment around the ligand-binding cleft corresponded to *Sat2-min* and *Sat2-off*, whereas *Retrosat2* and *RIRE8* show the most variable residue composition in its ligand-binding cavity/cleft (Figure 8D, Figure S6D).

The variability of the ligand-binding pockets reflects differential functional features of proteins. We have shown examples of possible ligands and/or cofactors that different ORF0 protein members can hold, which interactions drive the course of reactions, since different enzymatic activities has been exhibited by these ORF0 protein members, i.e. *Sat2-ruf*, *Sat2-pun*, and *FRetro3* can bind 2-(acetylamino)-2-deoxy-α-D-Glucopyranose (NDG); *Retrosat2*, *Sat2-off*, *RIRE8*, and *BraSat2* as example of interaction with cofactors as Co, Zn, SO4, and Fe2/S2 respectively (Figure 8E, Figures S3, S4, S5, S6E). The residue conservation of the ligand/cofactor binding sites and structural comparisons of the ligand-dependent regions inside of the ORF0 protein members are crucial for predicting the cofactor/ligand specificity and the enzymatic mechanism and possible functional characteristics.

We further conducted functional analysis of ORF0 by gene ontology annotations based on ORF0 protein structural information (Table S6). The structure-based functional inferences indicate that ORF0 protein members fall into three groups: Group 1 consists in the class 3 hydrolases (EC 3.1) acting on ester bonds, and includes *Sat2-min*, *Sat2-pun*, *Sat2-ruf*, *Retrosat2*, *Sat2-off*, *FRetro3*, *RIRE3*, *RIRE8*, and *ZmSat2*. Group 2 corresponding to the acyltransferases (EC 2.3) includes *SorSat2* (EC 2.3.1 Transferring groups other than amino-acyl groups are implicated in the biosynthesis of lipids. Group 3 of the small molecules transporters (EC 2.A), which includes *BraSat2*, are implicated in the antiport of glycerol-P (Table S6). The Group 1 can be further differentiated into four functional subgroups. Subgroup 1 includes *Sat2-min*, *Sat2-pun*, *Sat2-ruf* for which the molecular function is Carboxylic (Triacylglycerol) ester hydrolase (EC 3.1.1) implicated in lipid catabolic processes. Subgroup 2 includes *ZmSat2* and is implicated in removing 5′-nucleotides from 3′-hydroxyterminated oligonucleotides (EC 3.1.4 or Phosphoric-diester hydrolases). Subgroup 3 which includes *RIRE8* is implicated in DNA nicking (endodeoxyribonuclease) activity (EC 3.1.21). Subgroup 4, including *Sat2-off*, *RIRE3*, *Retrosat2* and *FRetro3* shares the same molecular function such as arylsulfatase activity (EC 3.1.6), which could be implicated in metabolic processes of polysaccharides.

## Discussion

### The evolutionary impacts of the longer LTR of *Retrosat2*

LTRs are critical for retrotransposon replication because the regions harbor regulatory signals necessary for gene expression. Unlike the coding regions of retrotransposons, LTRs are extremely variable in size ranging in size from 80 bp [16], [17] to more than 5 kb [18]. In many organisms, the LTRs are relative short (<1.5–2.0 kb). For instance, LTRs over 1.5 kb have not been found in Drosophila, yeast or *Anopheles gambiae* [41]–[43]. In plants, the LTRs of a majority of retrotransposon families are smaller than 2.0 kb [44]–[45]. Retrotransposons with LTRs greater than 3-kb tend to be uncommon.

It is not evident why elements with short LTRs would predominate genomes. Previous results indicated that increased length of LTRs dramatically reduces transcription efficiency and inhibits transposition [46]. Thus, elements with short LTRs may have a higher transposition frequency than those with longer LTRs. The LTRs of *Retrosat2* are over 3.2 kb, one of the longest LTRs in rice [45], and yet we show that Retrosat2 is abundant and may still be active in rice.

We identified regulatory putative signals in *Retrosat2* LTRs, including cis-acting regulatory elements involved in stress responsiveness (Table S4). Some of the regulatory signals in the LTR are multicopy, such as the three polyadenylation motifs. Multiple regulatory motifs have been found in other retroelements. In barley, for instance, the LTR of the *BARE* retrotransposon contains two TATA-box motifs, TATA1 and TATA2, that control tissue-specific expression. Both promoters are active in callus but TATA2 is active in embryos, whereas, TATA1 is virtually inactive in embryos [47]. Although RT-PCR indicates that *Retrosat2* is expressed in leaf, sheath and flower tissues of rice, we do not know whether the retroelement is expressed in other tissues or how the mulitple regulatory signals may affect expression patterns.

Divergence or mutation of LTR sequences may affect the activity of a retrotransposon. It has been reported that even a 10-bp sequence divergence in the U3 region can result in significant differences in promoter activities [10]. The LTRs of *Retrosat2* are more than 3.2 kb and contain multi-copy regulatory signals. Thus, it is possible that the element can accommodate mutations within LTRs due to redundancy of regulatory sequences without losing activity. We hypothesize that elements with long LTRs and multicopy regulatory sequences would be more tolerant of mutations than shorter LTRs with single copy signals.

### The genomic distributions of *Retrosat2* and the homologs

LTR retrotransposon families in plants have distinct chromosomal distribution patterns. Some LTR retrotransposons are predominantly found in intergenic regions, whereas, others are concentrated in heterochromatic regions [23]. Elements within a family tend to have similar distribution patterns. For example, the rice LTR retroelement *Dasheng* is related to *Gran3* from wild rice, *O. granulata*, and both elements are concentrated in centromeric and pericentromeric regions [28], [48]–[49]. The CRs are also concentrated in the centromeric regions across the grass genomes including rice, wild rice, wheat, barley and maize [24]–[28]. However, our previous results showed that the canonical CRR (CR of rice) identified in centromeres of all other *Oryza* species is absent in *O. brachyantha* and that a new retrotransposon, *FRetro3*, colonized the centromeres of this species [29].

Sequence comparisons, phylogenetic analysis and structural and functional annotation of proteins indicate that *FRetro3* and *Retrosat2* share a common ancestor. Despite the evolutionary and structural relationship to *Fretro3*, however, *Retrosat2* is not concentrated in rice centromeric regions but is dispersed across the genome. In addition, homologs of both *FRetro3* and *Retrosat2* are dispersed throughout the genomes of maize, sorghum and others (data not shown). These observations suggest that homologous elements derived from a common ancestor can have distinct distribution patterns and therefore different roles in shaping host genomes.

Since maize and sorghum are more distantly related than *O. brachyantha* and rice, the distinct distribution and function of *FRetro3* likely emerged after the split of the *Oryza* genus. It is not clear why or even how *FRetro3* became the functional centromeric retroelement in *O. brachyantha*, although, we assume that selection on *FRetro3* in *O. brachyantha* differed from that on *Restrosat2* in Nipponbare that allowed *FRetro3* to be domesticated to take over the role of the cannonical CRs.

### The origin and functional divergence of the ORF0 sequences

*Retrosat2* contains an extra and unique ORF, ORF0, whose origin and function has not been previously described. We identified homologous elements of *Retrosat2* in wild rice species, maize and sorghum as well as Arabidopsis, soybean and *Selaginella moellendorffii*. These results suggest an ancient origin of *Retrosat2* where an ancestral element was present before the divergence of *S. moellendorffii*, dicotyledonous and monocotyledonous plants. We found that homologs in wild rice species, maize and sorghum all contain the ORF0 domain but it was not found outside the grass family. One explanation is that the ORF0 was present the ancestral element but was deleted in Arabidopsis, soybean and other species after the divergence of the grass family from the other lineages. It is more likely, however, that the ORF0 evolved after the radiation of the grass from the other organisms.

In rice, two other LTR retroelements, *RIRE3* and *RIRE8*, also contain ORF0 and ORF1 sequences [50] and are present in other *Oryza* species (Table S3). *Retrosat2* likely shared a common ancestor with *RIRE3* and *RIRE8* based on the following observations: 1) *Retrosat2* is structurally similar to *RIRE3* and *RIRE8*, all three contain longer LTRs (∼3 kb), ORF0 and ORF1 sequences; 2) LTRs of both *RIRE3* and *RIRE8* show sequence identity with *Retrosat2*, and the 10-bp inverted terminal repeats of LTRs from *RIRE8* and *Retrosat2* are identical (5′TGTCACACCC–GGGCGTGACA3′); 3) ORF0 and ORF1 proteins of *RIRE3* and *RIRE8* share 30–34% and 69–70% similarity with *Retrosat2*, respectively; and 4) Phylogenetic trees based on both ORF0 and RTs of ORF1 show an evolutionary relationship between *RIRE3*, *RIRE8* and *Retrosat2* (Figure 6A,B).

A possible evolutionary scenario would be that the ancestral *Retrosat2* element and homologs contained one ORF that encoded a retrotransposase. Sometime between the separation of dicotyledonous and monocotyledonous plants (about 200 MYA) and the formation of the grasses (∼50–80 MYA), ORF0 evolved via a frameshift mutation or through capture of some unrelated DNA sequence. The divergence of *Retrosat2*, *RIRE3* and *RIRE8* likely occurred before the divergence of the *Oryza* genus (10–20 MYA) as the three families are detected in all 12 genomes comprising the *Oryza* genus (Table S3).

The ORF1 sequences of *Retrosat2* and homologs encode all the enzymes necessary for retrotransposition, thus, there is no obvious function for the ORF0. An amino acid sequence alignment of the ORF0 members reveals a wide range of sequence identities (7.6 to 90.2%) which is also reflected in the overall folding pattern of the ORF0 protein members. In a few cases, some of the predicted structures do share a common fold (*Sat2-min*, *Sat2-pun*, and *Sat2-ruf*) with discernible domains in each monomeric subunit, showing differences in the 2D structural elements. Observed differences in organization of these domains among the various ORF0 proteins reflects intrinsic features of structural and functional protein divergences. Comparative analyses of protein electrostatic potentials and structural modeling are key tools for enzyme classification and functional characterization. Electrostatic potentials of ORF0 enzymes allowed us to organize them and compare possible functional differences. The 3D structural and the functional analysis of ORF0 by gene ontology annotations suggest that the ORF0s encode enzymes implicated in metabolism (catabolic processes) with hydrolyses activities, but it is not clear how the ORF0 may affect transposition of *Retrosat2*. Moreover, we identified specific protein surface interaction properties (protein-protein, protein-cofactor and/or protein-substrate interactions) in different domains of the ORF0 protein members.

We observed differential abundance and distribution of positive and negative charges in the protein surfaces, which might correlate with differences or similarities in their activity, ligand-binding or protein-protein interactions [51], as well as differences in the mechanism of action and/or interaction with other proteins and intracellular targets [52]. This differential distribution could directly affect the interaction of the protein with other partners and target it to different sub-cellular localizations. Functional differences are also reflected in the surfaces of the ligand-binding domains and the cofactor-binding domains, which are more different in electrostatic charge distributions inside the ORF0 protein members.

Multiple regulatory signals were found in LTRs of *Retrosat2* and homologs were found in dicotyledons, monocotyledons and *S. moellendorffii* which suggest ancient origin of this retroelement family. Unlike CRs and other retrotransposons, members of *Retrosat2* family had distinct chromosomal/genomic distribution patterns that varied by species. Structural and functional divergence of ORF0s was seen via 3D predictions of ORF0 proteins and comparisons of their electrostatic surfaces, structural and key functional residues. In addition, the ORF0s likely encode enzymes implicated in metabolism (catabolic processes). Our data provide insight into the structural and functional features of ORF0 proteins and indicates that the genomic distribution and evolutionary functions of *Retrosat2* homologs, derived from an ancient retrotransposon family, have been subjected to unique selection pressures in their host genomes that have resulted in varied evolutionary trajectories.

## Materials and Methods

### Genome Database

The genome sequence of Nipponbare (*Oryza sativa* ssp. japonica) was downloaded from the International Rice Genome Sequencing Project (IRGSP) website (http://rgp.dna.affrc.go.jp/E/IRGSP/index.html). The draft genome sequence of 93–11 was downloaded from the BGI website (http://rice.genomics.org.cn/rice/link/download.jsp). Other genome sequences, including maize, sorghum, *Brachypodium*, Arabidopsis, papaya, soybean, wine grape poplar and *Selaginella moellendorffii*, were obtained from the PlantGDB website (http://www.plantgdb.org/prj/GenomeBrowser).

### Southern blot analysis

Young leaves from Nipponbare and other 13 rice species, including *O. glaberrima* (AA), *O. nivara* (AA), *O. longistaminata* (AA), *O. rufipogon* (AA), *O. punctata* (BB), *O. minuta* (BBCC), *O. officinalis* (CC), *O. alta* (CCDD), *O. australiensis* (EE), *O. brachyantha* (FF), *O. granulata* (GG), *O. ridleyi* (HHJJ) and *O. coarctata* (HHKK), were used to extract the genomic DNAs. 6 µg genomic DNAs of all 14 rice species were treated by *EcoRI* enzyme (Invitrogen, Carlsbad, CA) at 37 C for 10 h. Digested DNAs were separated by electrophoresis on a 1.0% (w/v) agarose gel at 55 V for 11 h, and blotted onto Hybond N+ membrane (Amersham Biosciences, now part of GE Life Sciences). The LTR sequence of *Retrosat2* was used as a probe to hybrid the genomic DNAs from 14 rice species. The PCR product was amplified using Nipponbare DNA as the template with the primers (forward: 5′ TGTGGAATTTTCCTTGAGTT3′; reverse: 5′ GAGTGGGGAGGAGAGAGA-3′), and then was labeled with [^32^P] dCTP using the rediprime II random prime labeling system (Amersham Biosciences). Hybridizations were performed at 56°C for overnight and washed in 1.5×SSC/0.1% SDS solution for 35 min and in 1×SSC/0.1% SDS solution for 35 min. The membrane was exposed on a Fuji-image plate for 36 h, and the hybridization signals were captured using a Fujifilm FLA scanner.

### RT-PCR

Total RNA was isolated from leaves, sheaths of 4-week old plants and young spikes (3–5 cm) of Nipponbare using the TRIZOL Reagent (Invitrogen, Carlsbad, CA). Five microgram RNA from each sample was converted into single-strand cDNA with reverse transcriptase (Invitrogen, Carlsbad, CA) according to the manufacture’s recommendations. Reactions were diluted 4- to 5-fold, and 2 µl of the diluted cDNAs were used as templates for PCR amplifications with the forward primer (5′-TGCCCTGGAAGAACTTATCG-3′) and reverse primer (5′ ACCACACCTCAGGTTTCACC-3′) which target the U5 region of *Retrosat2* LTRs. The rice actin gene (*Os03g0718100*) was amplified in parallel with *Retrosat2* as the control for quantitative comparison of mRNA levels, using actinF (5′-CAAGGCCAATCGTGAGAA-3′) and actinR (5′-AGCAATGCCAGGGAACATAGT-3′) primers. All plants of Nipponbare were grown in a greenhouse at University of Georgia.

### Fluorescence *in situ* hybridization (FISH) analysis

Slide preparation and FISH were conducted following published protocols [53]. Briefly, young panicles of *Oryza sativa* L. ssp. japonica cv. Nipponbare were harvested and fixed in 3∶1 ethanol and glacial acetic acid for 24 hrs at room temperature, and then stored at 4°C. Pachytene chromosomes were prepared by squashing anthers in acetic acid. Slides were stored at −80°C until use. The plasmid clone containing the rice specific satellite repeat CentO (GenBank accession: AF058902) was provided by Drs. Jiming Jiang and Jason Walling at University of Wisconsin and used to determine the locations of centromeres. A pair of PCR primers (Forward: 5′-GCTCCGTTTAATCCCATTCA-3′, Reverse: 5′-TGTATTAAAACCCCCGTCCA-3′) were used to amplify the LTR sequence of *Retrosat2* from Nipponbare that was then used as a probe for FISH. CentO and *Retrosat2* were nick translated with biotin dUTP or digoxigenin dUTP (Roche), and visualized with Streptavidin Alexa Fluor 488 (Invitrogen) or Anti-digoxigenin-rhodamine (Roche), respectively. The chromosome images were captured with a Nikon Eclipse 80i microscope (http://www.nikon.com), equipped with a Photometrics CoolSnap HQ CCD camera (http://www.photometrics.com), controlled with MetaVue imaging software (http://www.moleculardevices.com/). Adobe Photoshop CS3 (Adobe Systems Incorporated) was used to produce publication images.

### Phylogenetic analysis and calculation of Ka/Ks values of ORF0 and ORF1 sequences

The ORF0 and ORF1 sequences of *Retrosat2* and other LTR retrotransposons were used to generate multiple alignments using CLUSTALW (http://www.ebi.ac.uk/clustalw) with default options. Phylogenetic trees were generated using the neighbor-joining method with the MEGA4 program (http://www.megasoftware.net). A total of 37 LTR retrotransposons were used to construct the phylogenetic trees, including *Retrosat2* (AF111709), *RIRE2* (AB030283), *RIRE3* (AB014738), *RIRE7* (AB033235), *RIRE8* (AB014740) and other seven rice LTR retroelements (*Osr28*, *Osr30*, *Osr35*, *Osr38*, *Osr39*, *Osr40* and *Osr42* [54]), four elemements (*OGRetro3*, *OGRetro9*, *OGRetro23* and *OGRetro52*) in *O. glanulata* [28], *Sat2-ruf* in *O. rufipogon* (FJ581045), *Sat2-pun* in *O. punctata* (AC215214), *Sat2-min* in *O. minuta* (AC232156), *Sat2-off* in *O. officinalis* (AC240793), *dingo* in *O. australiensis* [30], *FRetro3* in *O. brachyantha* [29], *Reina* (U69258) and CRM (AY129008) in maize, *Retrosor1* (AF098806) in sorghum, *cereba* (AY040832) in barley, *Athena* in Arabidopsis (AC007209), *Cft1* in *Cladosporium fulvum* (Z11866) and *gypsy* in Drosophila (M12927). The phylogenetic analysis was conducted based on 1000 bootstrap replicates. The synonymous (Ks) and non-synonymous (Ka) substitution rates of the ORF0 and ORF1 sequences were calculated using the PAL2NAL program [55].

### Homology modeling

To understand and compare the structural and molecular conformation between proteins, all sequences were modeled using the top ten PDB closed template structures by SWISS-MODEL, a protein structure homology-modeling server, via the ExPASy web server [56]–[58]. Several models were generated and evaluated. The different protein models were subjected to energy minimization with GROMOS96 force field energy [59] implemented in DeepView/Swiss-PDBViewer v3.7 [60] to improve the van der Waals contacts and correct the stereochemistry of the model. Stereochemical quality of the selected model was evaluated using PROCHECK [61], PROSA [62] and WHATCHECK [63] programs, as well as the protein energy with ANOLEA [64]. The Ramachandran plot statistics for the models were calculated to show the number of protein residues in the favored regions. Every protein model was superimposed on the best template crystal structure, as well as structural comparisons between them to calculate average distance between their Cα backbones. Protein superimpositions and surface protein contours analysis were performed and visualized in PyMol software [65].

The theoretical model was submitted to ConSurf server [66] in order to generate evolutionary related conservation scores aimed to the identification of functional region in proteins. Functional and structural key residues in the ORF0 sequences were confirmed by ConSeq server [67].

### Ligand-binding domains and function prediction based on the protein structures

Prediction of the best identified ligand-binding site or domain in the built structure was made by sequence and structure-based approaches to protein function inference and ligand screening. This approach use an algorithm for ligand binding site prediction, ligand screening and molecular function prediction, which is based on binding site conservation across evolutionary distant proteins identified by threading [68].

The identification of functional analogs of the query protein based on the built 3D models predicted in Gene Ontology (GO) terms, showing the molecular function and biological processes in which proteins are implicated, as well as Enzyme Classification (EC) numbers are good indicator of the functional similarity between the query and the identified enzyme analogs. This process was developed by using The Gene Ontology project describing fundamental characteristics of genes and their products [69].

### Electrostatic potentials

Electrostatic Poisson-Boltzmann (PB) potentials were obtained by using the APBS 1.0.0 plugging [70] for the molecular modeling software PyMol software (DeLano Scientific LLC), with AMBER99 [71] to assign the charges and radii to all of the atoms (including hydrogens), which were added and optimized with the Python software package PDB2PQR [72].

Fine grid spaces of 0.35 A° were used to solve the linearized PB equation in sequential focusing multigrid calculations in a mesh of 130 points per dimension at 310.00 K. The dielectric constants were two for the protein and 80.00 for water. The output mesh was processed in the scalar OpenDX format to render isocontours and maps onto the surfaces with PyMOL software. Potential values are given in units of kT per unit charge (k Boltzmann's constant; T temperature).

## Supporting Information

Figure S1 **RT-PCR of** ***Retrosat2*** **.** L, S, F means leaf, sheath and flower, respectively.
(TIF)

Figure S2 **Dot-plot of LTRs of** ***Retrosat2*** **and the homologs.**
(TIF)

Figure S3 **Detailed structural conformation and conservation analysis of ORF0 of** ***Sat2-off.*** (A) General structure (cartoon diagram) shows the superimposition of ORF0 of *Sat2-off* (white) and rice *RetroSat2* (blue), with a RMSD = 5.693Å calculated for the superimposition of structural carbon α. Represented structures were rotated at 180u. (B) The surface conformation of *Sat2-off* (rotated 180u) showing the secondary structure elements inside is depicted. (C) Electrostatic surface potential showing front, back, top and bottom views of *Sat2-off* structure. The surface colors are clamped at red (−10) or blue (+10). Top and bottom views are highlighted with a white line coming from front view. (D) Best predicted *Sat2-off* model (2D-structure) was subject to consurf-conservational analysis searching for close homologous sequences with known structures using PSI-BLAST. The protein was finally visualized using FirstGlance in Jmol with the conservation scores being color-coded. The conserved and variable residues are presented as space-filled models and colored according to the conservation scores. A detailed view of the predicted ligand-binding cavity holding up the cofactor/ligand (van der Walls spheres and/or lines) is shown in high magnification. Represented structures were rotated at 180u. (E) Cartoon structural representation of a general front view of *Sat2-off* model (C- and N-terminal colored as blue and red respectively), showing the morphology of the predicted cofactor/ligand-binding pocket/cavity. A detailed view at higher magnification is highlighting the residues implicated in this cavity formation and interaction with the ligand SO_4_, which are D138, H141, R146, and A160.
(TIF)

Figure S4 **Detailed structural conformation and conservation analysis of** ***FRetro3*** **, a rice ORF0 protein member.** (A) General structure (cartoon diagram rainbow colored) shows the 2D structural elements of the rice *FRetro3*, where N- and C-terminal are colored blue and red respectively. Represented structures were rotated at 180u. (B) The surface conformation of *FRetro3* (rotated 180u) showing the secondary structure elements inside is depicted. (C) Electrostatic surface potential showing front, back, top and bottom views of *FRetro3* structure. The surface colors are clamped at red (−10) or blue (+10). Top and bottom views are highlighted with a white line coming from front view. (D) Best predicted *FRetro3* model (2D-structure) was subject to consurf-conservational analysis searching for close homologous sequences with known structures using PSI-BLAST. The protein was finally visualized using FirstGlance in Jmol with the conservation scores being color-coded. The conserved and variable residues are presented as space-filled models and colored according to the conservation scores. A detailed view of the predicted ligand-binding cavity holding up the cofactor/ligand (van der Walls spheres and/or lines) is shown in high magnification. Represented structures were rotated at 180u. (E) Cartoon structural representation of a general front view of *FRetro3* model (C- and N-terminal colored as blue and red respectively), showing the morphology of the predicted cofactor/ligand-binding pocket/cavity. A detailed view at higher magnification is highlighting the residues implicated in this cavity formation and interaction with the ligand 2-(acetylamino)-2-deoxy-α-D-Glucopyranose (NDG), which are F13, V37, L38, R39, A53, E54, A67, and A87.
(TIF)

Figure S5 **Detailed structural conformation and conservation analysis of ORF0 sequence of** ***BraSat2.*** (A) General structure (cartoon diagram rainbow colored) shows the 2D structural elements of the ORF0 of *BraSat2*, where N- and C-terminal are colored blue and red respectively. Represented structures were rotated at 180u. (B) The surface conformation of *BraSat2* (rotated 180u) showing the secondary structure elements inside is depicted. (C) Electrostatic surface potential showing front, back, top and bottom views of *BraSat2* structure. The surface colors are clamped at red (−10) or blue (+10). Top and bottom views are highlighted with a white line coming from front view. (D) Best predicted *BraSat2* model (2D-structure) was subject to consurf-conservational analysis searching for close homologous sequences with known structures using PSI-BLAST. The protein was finally visualized using FirstGlance in Jmol with the conservation scores being color-coded. The conserved and variable residues are presented as space-filled models and colored according to the conservation scores. A detailed view of the predicted ligand-binding cavity holding up the cofactor/ligand (van der Walls spheres and/or lines) is shown in high magnification. Represented structures were rotated at 180u. (E) Cartoon structural representation of a general front view of *BraSat2* model (C- and N-terminal colored as blue and red respectively), showing the morphology of the predicted cofactor/ligand-binding pocket/cavity. A detailed view at higher magnification is highlighting the residues implicated in this cavity formation and interaction with the ligand FE2/S2 (inorganic) cluster (FES), which are A10, G11, Q12, D13, L15, F17, E18, Q20, and P60.
(TIF)

Figure S6 **Detailed structural conformation and conservation analysis of** ***RIRE8*** **, a rice ORF0 protein member.** (A) General structure (cartoon diagram rainbow colored) shows the 2D structural elements of the rice *RIRE8*, where N- and C-terminal are colored blue and red respectively. Represented structures were rotated at 180u. (B) The surface conformation of *RIRE8* (rotated 180u) showing the secondary structure elements inside is depicted. (C) Electrostatic surface potential showing front, back, top and bottom views of *RIRE8* structure. The surface colors are clamped at red (−10) or blue (+10). Top and bottom views are highlighted with a white line coming from front view. (D) Best predicted RIRE8 model (2D-structure) was subject to consurf-conservational analysis searching for close homologous sequences with known structures using PSI-BLAST. The protein was finally visualized using FirstGlance in Jmol with the conservation scores being color-coded. The conserved and variable residues are presented as space-filled models and colored according to the conservation scores. A detailed view of the predicted ligand-binding cavity holding up the cofactor/ligand (van der Walls spheres and/or lines) is shown in high magnification. Represented structures were rotated at 180u. (E) Cartoon structural representation of a general front view of *RIRE8* model (C- and N-terminal colored as blue and red respectively), showing the morphology of the predicted cofactor/ligand-binding pocket/cavity. A detailed view at higher magnification is highlighting the residues implicated in this cavity formation and interaction with the ligand cobalt (Co), which are E121, T124, D143, and D148.
(TIF)

Table S1 **The** ***Retrosat2*** **element in Nipponbare genome**.
(DOC)

Table S2 **Homologous elements of** ***Retrosat2*** **in** ***Oryza*** **genus.**
(DOC)

Table S3 **Distributions of** ***Retrosat2*** **,** ***RIRE3*** **and** ***RIRE8*** **families in BESs of 12 genomes.**
(DOC)

Table S4 **Regulatory signals in the LTR region of** ***Retrosat2*** **.**
(DOC)

Table S5 **Structural similarity among selected ORF0 protein members.**
(DOC)

Table S6 **Functional characteristics of the ORF0 protein members based on structural features.**
(DOC)
