# Supplementary figures and images for: Functional and Structural Divergence of an Unusual LTR Retrotransposon Family in Plants
Source: PLoS One. 2012 Oct 31;7(10):e48595. doi: 10.1371/journal.pone.0048595 (PMC3485330; doi:10.1371/journal.pone.0048595)

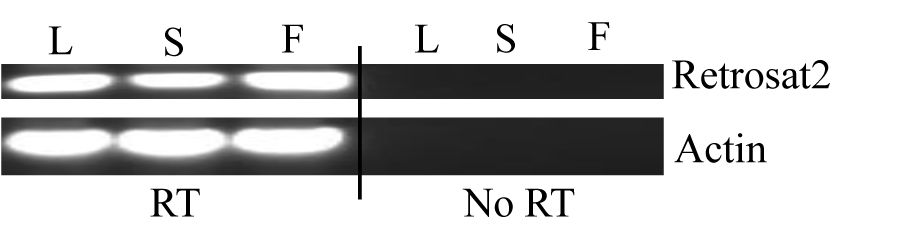

Supplement: Figure S1 — RT-PCR of Retrosat2 . L, S, F means leaf, sheath and flower, respectively. (TIF) [file pone.0048595.s001.tif]

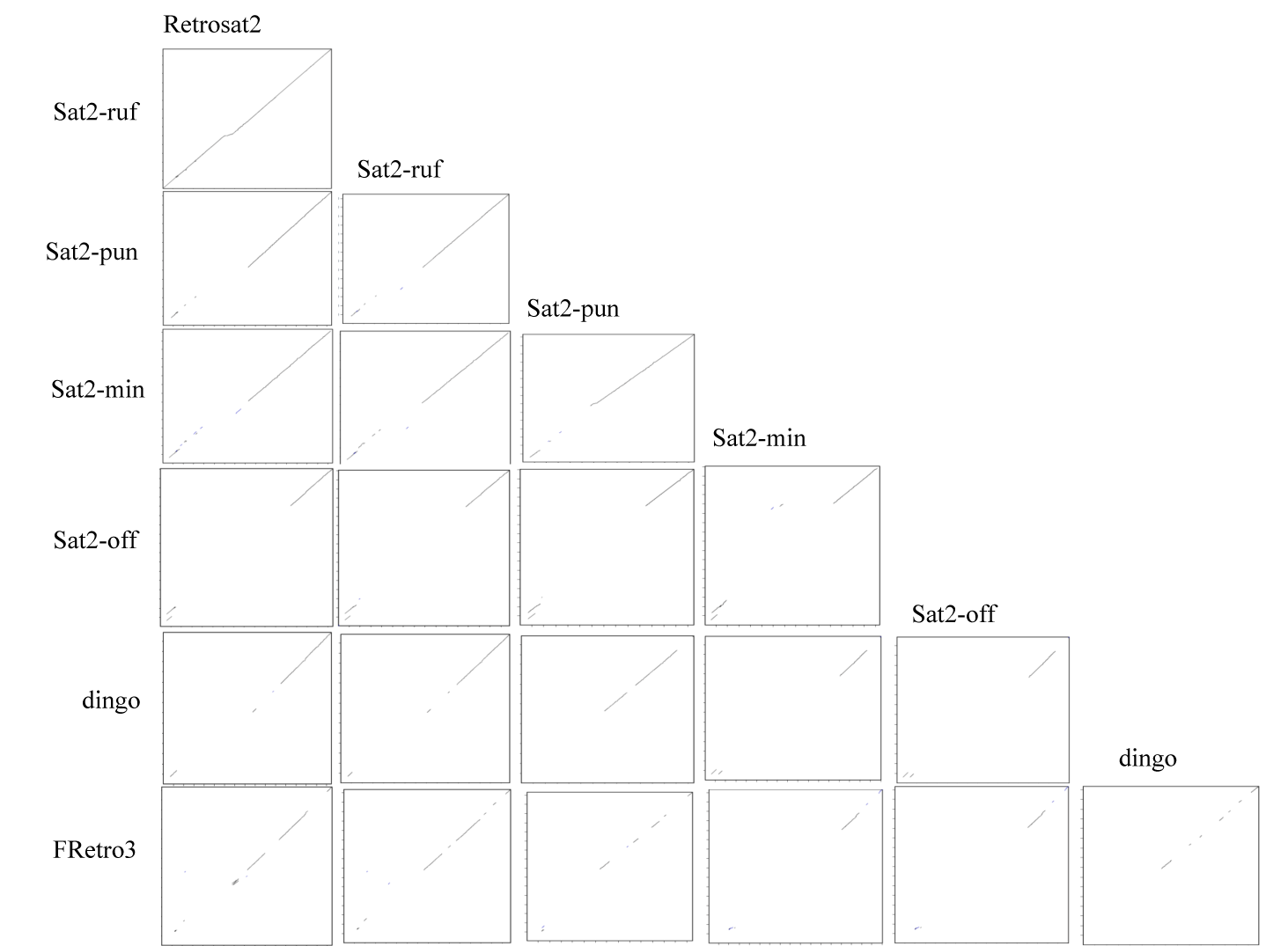

Supplement: Figure S2 — Dot-plot of LTRs of Retrosat2 and the homologs. (TIF) [file pone.0048595.s002.tif]

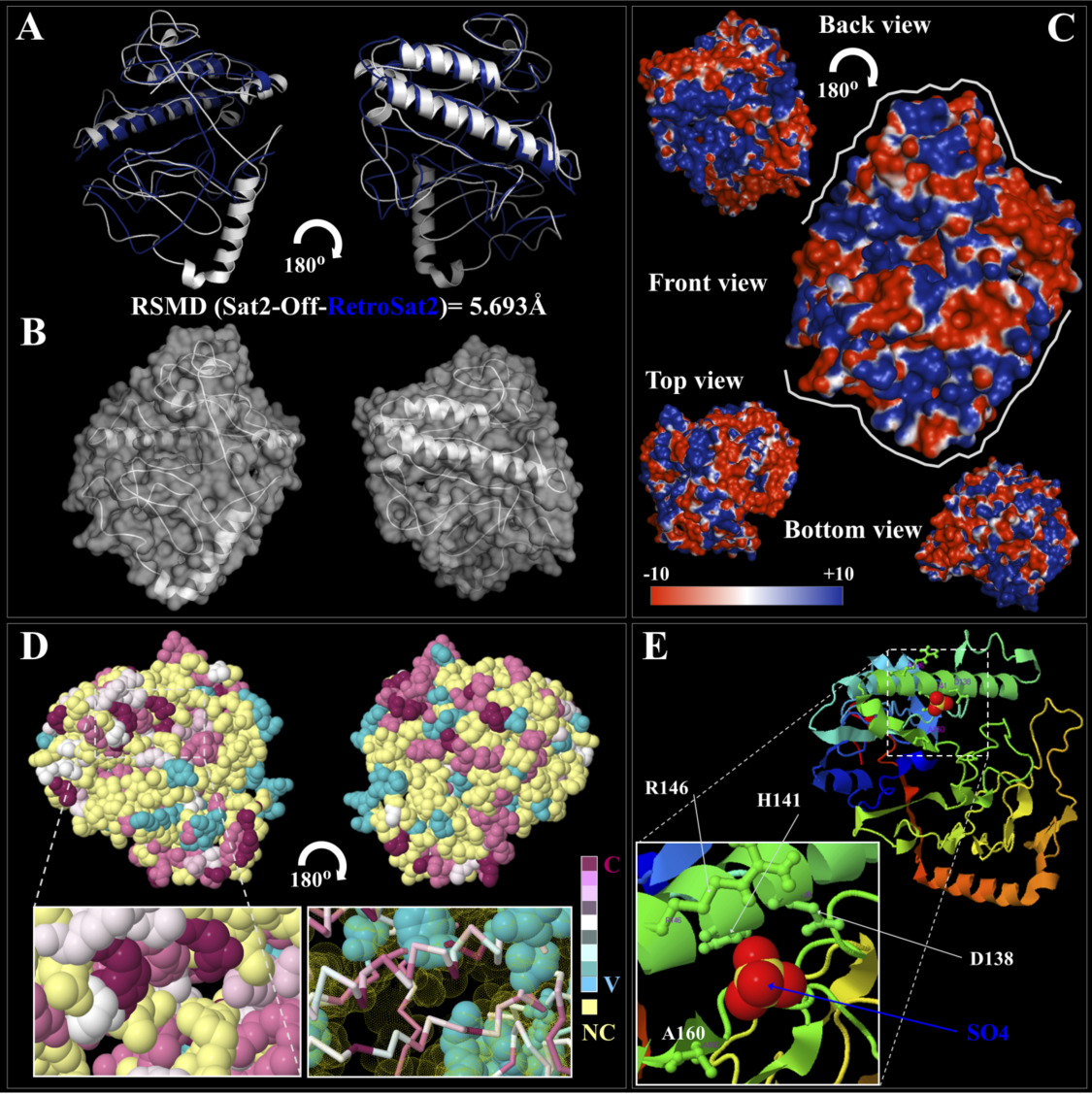

Supplement: Figure S3 — Detailed structural conformation and conservation analysis of ORF0 of Sat2-off. (A) General structure (cartoon diagram) shows the superimposition of ORF0 of Sat2-off (white) and rice RetroSat2 (blue), with a RMSD = 5.693Å calculated for the superimposition of structural carbon α. Represented structures were rotated at 180u. (B) The surface conformation of Sat2-off (rotated 180u) showing the secondary structure elements inside is depicted. (C) Electrostatic surface potential showing front, back, top and bottom views of Sat2-off structure. The surface colors are clamped at red (−10) or blue (+10). Top and bottom views are highlighted with a white line coming from front view. (D) Best predicted Sat2-off model (2D-structure) was subject to consurf-conservational analysis searching for close homologous sequences with known structures using PSI-BLAST. The protein was finally visualized using FirstGlance in Jmol with the conservation scores being color-coded. The conserved and variable residues are presented as space-filled models and colored according to the conservation scores. A detailed view of the predicted ligand-binding cavity holding up the cofactor/ligand (van der Walls spheres and/or lines) is shown in high magnification. Represented structures were rotated at 180u. (E) Cartoon structural representation of a general front view of Sat2-off model (C- and N-terminal colored as blue and red respectively), showing the morphology of the predicted cofactor/ligand-binding pocket/cavity. A detailed view at higher magnification is highlighting the residues implicated in this cavity formation and interaction with the ligand SO4, which are D138, H141, R146, and A160. (TIF) [file pone.0048595.s003.tif]

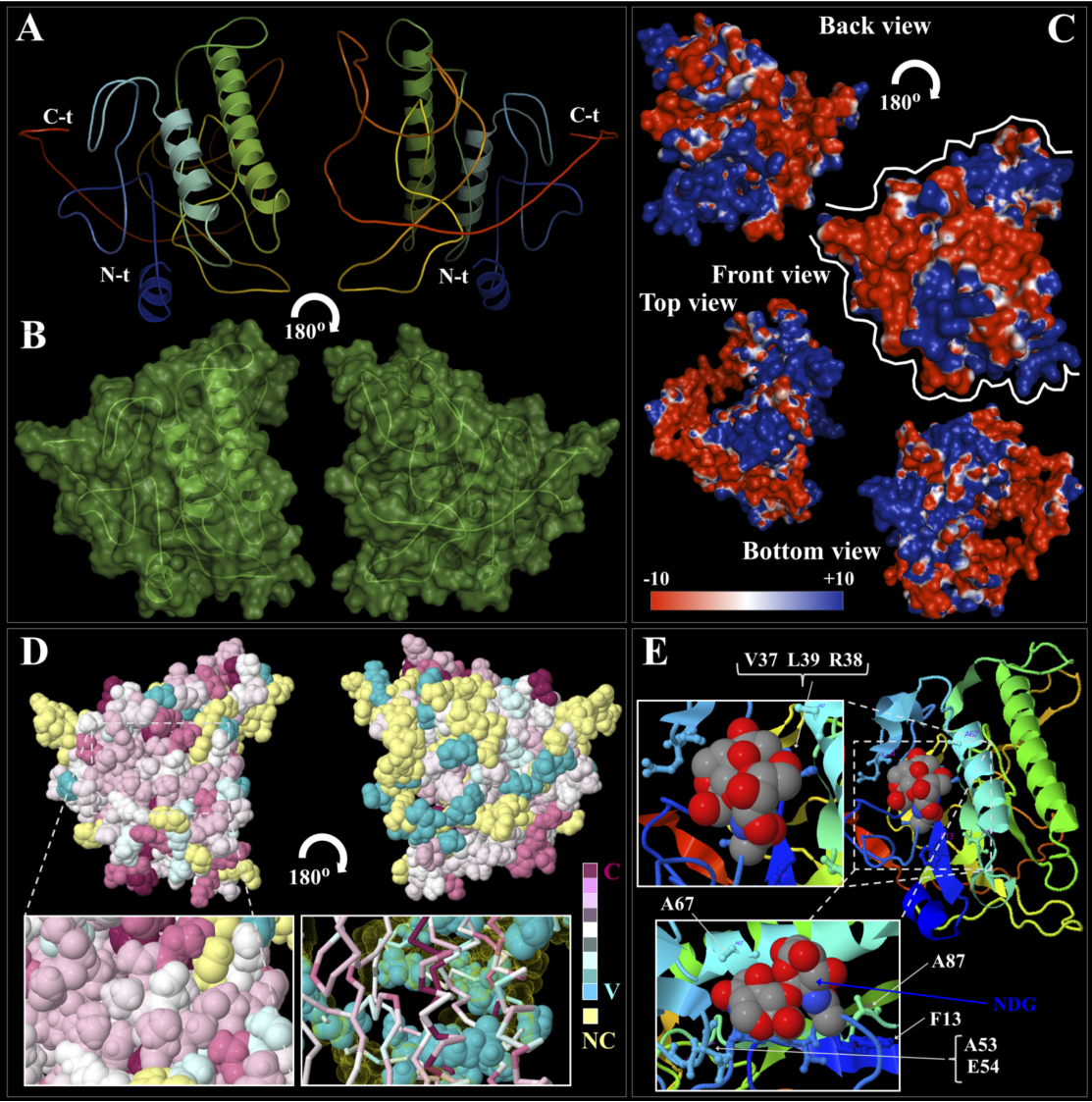

Supplement: Figure S4 — Detailed structural conformation and conservation analysis of FRetro3 , a rice ORF0 protein member. (A) General structure (cartoon diagram rainbow colored) shows the 2D structural elements of the rice FRetro3, where N- and C-terminal are colored blue and red respectively. Represented structures were rotated at 180u. (B) The surface conformation of FRetro3 (rotated 180u) showing the secondary structure elements inside is depicted. (C) Electrostatic surface potential showing front, back, top and bottom views of FRetro3 structure. The surface colors are clamped at red (−10) or blue (+10). Top and bottom views are highlighted with a white line coming from front view. (D) Best predicted FRetro3 model (2D-structure) was subject to consurf-conservational analysis searching for close homologous sequences with known structures using PSI-BLAST. The protein was finally visualized using FirstGlance in Jmol with the conservation scores being color-coded. The conserved and variable residues are presented as space-filled models and colored according to the conservation scores. A detailed view of the predicted ligand-binding cavity holding up the cofactor/ligand (van der Walls spheres and/or lines) is shown in high magnification. Represented structures were rotated at 180u. (E) Cartoon structural representation of a general front view of FRetro3 model (C- and N-terminal colored as blue and red respectively), showing the morphology of the predicted cofactor/ligand-binding pocket/cavity. A detailed view at higher magnification is highlighting the residues implicated in this cavity formation and interaction with the ligand 2-(acetylamino)-2-deoxy-α-D-Glucopyranose (NDG), which are F13, V37, L38, R39, A53, E54, A67, and A87. (TIF) [file pone.0048595.s004.tif]

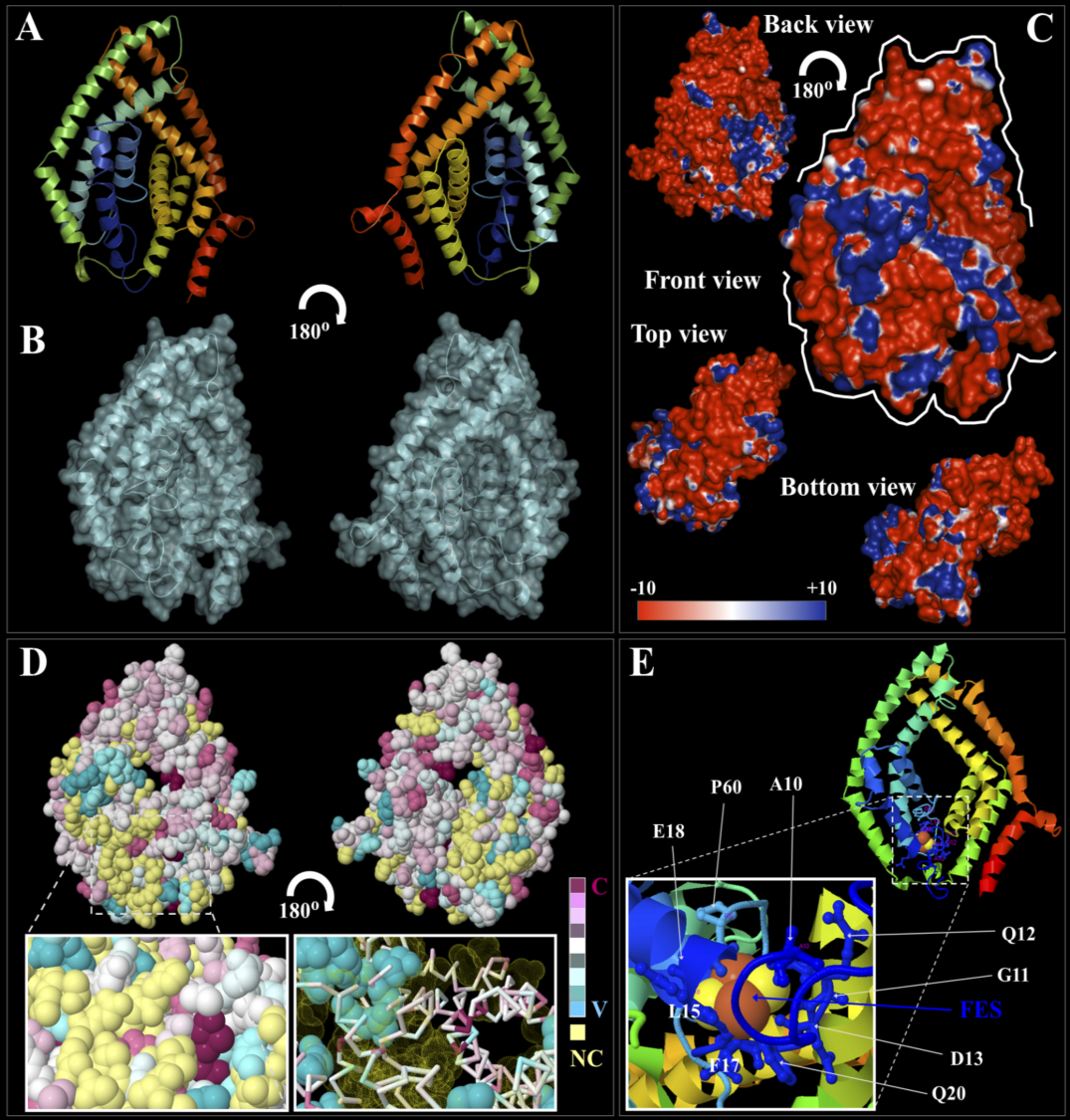

Supplement: Figure S5 — Detailed structural conformation and conservation analysis of ORF0 sequence of BraSat2. (A) General structure (cartoon diagram rainbow colored) shows the 2D structural elements of the ORF0 of BraSat2, where N- and C-terminal are colored blue and red respectively. Represented structures were rotated at 180u. (B) The surface conformation of BraSat2 (rotated 180u) showing the secondary structure elements inside is depicted. (C) Electrostatic surface potential showing front, back, top and bottom views of BraSat2 structure. The surface colors are clamped at red (−10) or blue (+10). Top and bottom views are highlighted with a white line coming from front view. (D) Best predicted BraSat2 model (2D-structure) was subject to consurf-conservational analysis searching for close homologous sequences with known structures using PSI-BLAST. The protein was finally visualized using FirstGlance in Jmol with the conservation scores being color-coded. The conserved and variable residues are presented as space-filled models and colored according to the conservation scores. A detailed view of the predicted ligand-binding cavity holding up the cofactor/ligand (van der Walls spheres and/or lines) is shown in high magnification. Represented structures were rotated at 180u. (E) Cartoon structural representation of a general front view of BraSat2 model (C- and N-terminal colored as blue and red respectively), showing the morphology of the predicted cofactor/ligand-binding pocket/cavity. A detailed view at higher magnification is highlighting the residues implicated in this cavity formation and interaction with the ligand FE2/S2 (inorganic) cluster (FES), which are A10, G11, Q12, D13, L15, F17, E18, Q20, and P60. (TIF) [file pone.0048595.s005.tif]

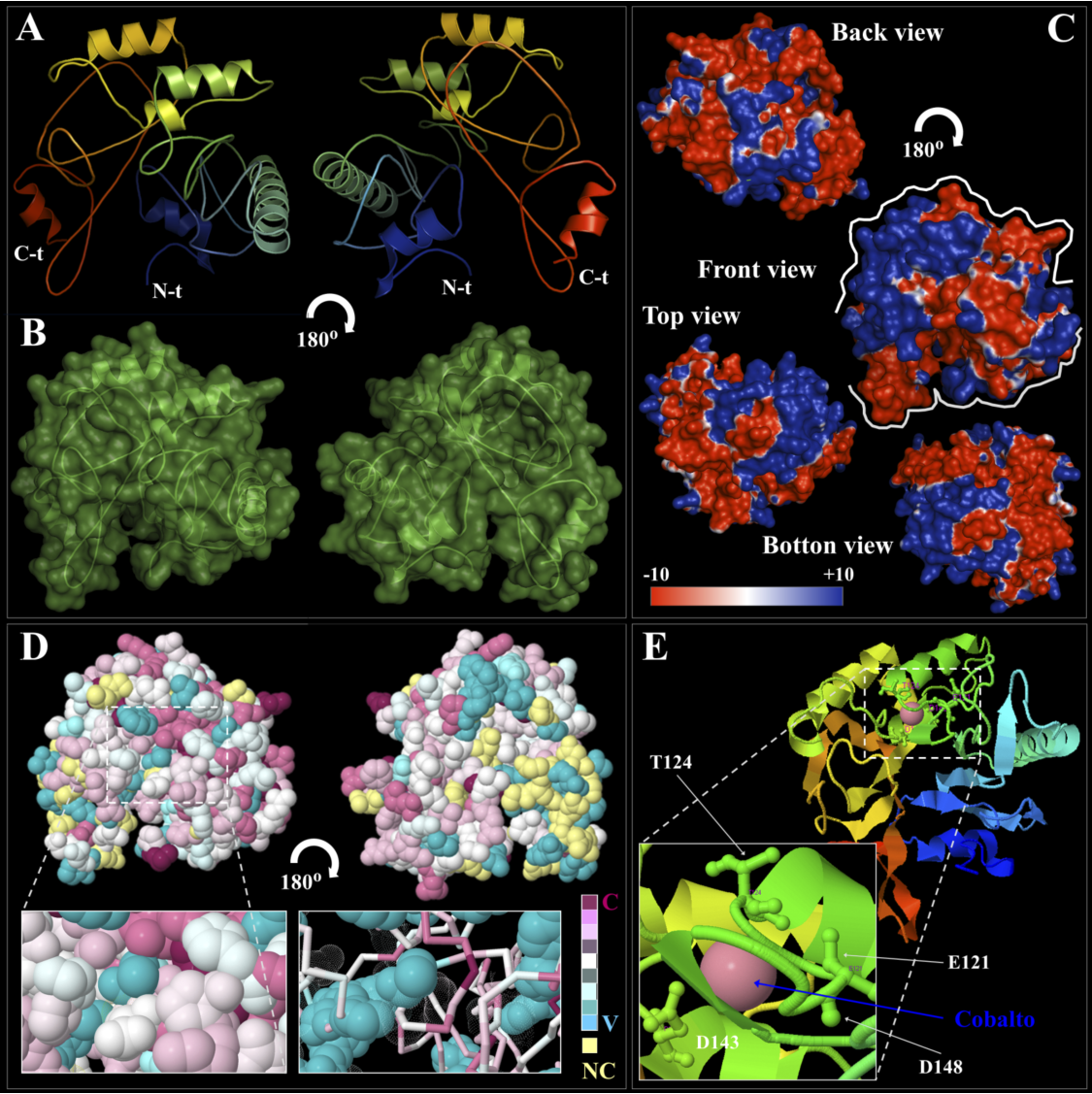

Supplement: Figure S6 — Detailed structural conformation and conservation analysis of RIRE8 , a rice ORF0 protein member. (A) General structure (cartoon diagram rainbow colored) shows the 2D structural elements of the rice RIRE8, where N- and C-terminal are colored blue and red respectively. Represented structures were rotated at 180u. (B) The surface conformation of RIRE8 (rotated 180u) showing the secondary structure elements inside is depicted. (C) Electrostatic surface potential showing front, back, top and bottom views of RIRE8 structure. The surface colors are clamped at red (−10) or blue (+10). Top and bottom views are highlighted with a white line coming from front view. (D) Best predicted RIRE8 model (2D-structure) was subject to consurf-conservational analysis searching for close homologous sequences with known structures using PSI-BLAST. The protein was finally visualized using FirstGlance in Jmol with the conservation scores being color-coded. The conserved and variable residues are presented as space-filled models and colored according to the conservation scores. A detailed view of the predicted ligand-binding cavity holding up the cofactor/ligand (van der Walls spheres and/or lines) is shown in high magnification. Represented structures were rotated at 180u. (E) Cartoon structural representation of a general front view of RIRE8 model (C- and N-terminal colored as blue and red respectively), showing the morphology of the predicted cofactor/ligand-binding pocket/cavity. A detailed view at higher magnification is highlighting the residues implicated in this cavity formation and interaction with the ligand cobalt (Co), which are E121, T124, D143, and D148. (TIF) [file pone.0048595.s006.tif]
