# Supplementary material for: Functional and Structural Divergence of an Unusual LTR Retrotransposon Family in Plants
Source: PLoS One. 2012 Oct 31;7(10):e48595. doi: 10.1371/journal.pone.0048595 (PMC3485330; doi:10.1371/journal.pone.0048595)
Supplement: Table S1 — The Retrosat2 element in Nipponbare genome. (DOC) [file pone.0048595.s007.doc]

Table S1. Retrosat2 elements in the Nipponbare genome

| Chromosome | | Intact element | | | Intact solo-LTR | | | Other element | | | All element | | |
| --- | --- | --- | --- | --- | --- | --- | --- | --- | --- | --- | --- | --- | --- |
| No. | Size  (bp) | Copy number | Coverage  (bp) | Percentage (100%) | Copy number | Coverage  (bp) | Percentage (100%) | Copy number | Coverage  (bp) | Percentage (100%) | Copy number | Coverage  (bp) | Percentage (100%) |
| 1 | 45038604 | 14 | 167836 | 0.37 | 29 | 94097 | 0.26 | 44 | 135199 | 0.36 | 87 | 397132 | 1.10 |
| 2 | 36792247 | 10 | 117229 | 0.32 | 34 | 104646 | 0.28 | 53 | 170242 | 0.46 | 97 | 392117 | 1.07 |
| 3 | 37312367 | 4 | 47273 | 0.13 | 42 | 130127 | 0.35 | 24 | 112376 | 0.30 | 70 | 289776 | 0.78 |
| 4 | 36060865 | 13 | 163938 | 0.45 | 43 | 132387 | 0.37 | 49 | 168613 | 0.47 | 105 | 464938 | 1.29 |
| 5 | 30073438 | 19 | 224184 | 0.75 | 33 | 90968 | 0.30 | 39 | 191281 | 0.64 | 91 | 506433 | 1.68 |
| 6 | 32124789 | 10 | 116538 | 0.36 | 33 | 100087 | 0.31 | 34 | 137556 | 0.43 | 77 | 354181 | 1.10 |
| 7 | 30357780 | 10 | 111905 | 0.37 | 42 | 130369 | 0.43 | 39 | 146139 | 0.48 | 91 | 388413 | 1.28 |
| 8 | 28530027 | 16 | 168396 | 0.59 | 46 | 140056 | 0.49 | 40 | 119716 | 0.42 | 102 | 428168 | 1.50 |
| 9 | 23895721 | 13 | 150756 | 0.63 | 33 | 102504 | 0.43 | 41 | 175300 | 0.73 | 87 | 428560 | 1.79 |
| 10 | 23703430 | 20 | 225247 | 0.95 | 34 | 101853 | 0.43 | 49 | 189854 | 0.80 | 101 | 516954 | 2.18 |
| 11 | 31219694 | 18 | 211531 | 0.68 | 29 | 85723 | 0.27 | 66 | 417576 | 1.33 | 113 | 491823 | 2.29 |
| 12 | 27679166 | 15 | 184865 | 0.67 | 31 | 96917 | 0.35 | 49 | 299082 | 1.08 | 95 | 580864 | 2.10 |
| Total | 382788128 | 162 | 1889698 | 0.49 | 429 | 1309734 | 0.34 | 527 | 2262934 | 0.46 | 1118 | 5462366 | 1.43 |
