# Supplementary material for: Functional and Structural Divergence of an Unusual LTR Retrotransposon Family in Plants
Source: PLoS One. 2012 Oct 31;7(10):e48595. doi: 10.1371/journal.pone.0048595 (PMC3485330; doi:10.1371/journal.pone.0048595)
Supplement: Table S2 — Homologous elements of Retrosat2 in Oryza genus. (DOC) [file pone.0048595.s008.doc]

Table S2. Homologous elements of Retrosat2 in the genus *Oryza*

| Name of homolog | Genome | BAC | Location in BAC | Size  (bp) | LTR size  (bp) |
| --- | --- | --- | --- | --- | --- |
| Sat2-ruf | *O. rufipogon* (AA) | FJ581045 | 213774-202019 | 11,756 | 3160 |
| Sat2-pun | *O. punctata* (BB) | AC215214 | 62464-49141 | 13,324 | 2897 |
| Sat2-min | *O. minuta* (BBCC) | AC232156 | 53685-41274 | 12,412 | 3068 |
| Sat2-off | *O. officinalis* (CC) | AC240793 | 218809-230873 | 12,065 | 2451-3406 |
| dingo | *O. australiensis* (EE) | DQ365822 | 1-11375 | 11,375 | 2399-2970 |
