# Supplementary material for: Functional and Structural Divergence of an Unusual LTR Retrotransposon Family in Plants
Source: PLoS One. 2012 Oct 31;7(10):e48595. doi: 10.1371/journal.pone.0048595 (PMC3485330; doi:10.1371/journal.pone.0048595)
Supplement: Table S3 — Distributions of Retrosat2 , RIRE3 and RIRE8 families in BESs of 12 genomes. (DOC) [file pone.0048595.s009.doc]

Table S3. Distribution of Retrosat2, RIRE3 and RIRE8 families in BESs of 12 *Oryza* genomes

| Species (genome type) | BES  size (bp) | Retrosat2 | | RIRE3 | | RIRE8 | |
| --- | --- | --- | --- | --- | --- | --- | --- |
|  |  | Coverage  (bp) | Fraction  (%) | Coverage  (bp) | Fraction  (%) | Coverage  (bp) | Fraction  (%) |
| *O. nivara* (AA) | 70550640 | 508297 | 0.72 | 1154513 | 1.64 | 2569230 | 3.64 |
| *O. rufipogon* (AA) | 49965990 | 308433 | 0.62 | 654299 | 1.31 | 1554185 | 3.11 |
| *O. glaberrima* (AA) | 39398047 | 110782 | 0.28 | 164464 | 0.42 | 351121 | 0.89 |
| *O. punctata* (BB) | 48571904 | 695064 | 1.43 | 118294 | 0.24 | 169380 | 0.35 |
| *O. minuta* (BBCC) | 94831544 | 1514740 | 1.60 | 184465 | 0.19 | 174782 | 0.18 |
| *O. officinalis* (CC) | 72478457 | 941559 | 1.30 | 145317 | 0.20 | 67500 | 0.09 |
| *O. alta* (CCDD) | 75490458 | 103994 | 0.14 | 119848 | 0.16 | 123245 | 0.16 |
| *O. australiensis* (EE) | 86979725 | 3237974 | 3.72 | 198349 | 0.23 | 316933 | 0.36 |
| *O. brachyantha* (FF) | 45300433 | 1089863 | 2.41 | 12596 | 0.03 | 4391 | 0.01 |
| *O. granulata* (GG) | 93172441 | 1373126 | 1.47 | 175046 | 0.19 | 982683 | 1.05 |
| *O. ridleyi* (HHJJ) | 129360456 | 745946 | 0.58 | 139924 | 0.11 | 103704 | 0.08 |
| *O. coarctata* (HHKK) | 129024847 | 42169 | 0.03 | 150938 | 0.12 | 129643 | 0.10 |
