# Supplementary material for: Functional and Structural Divergence of an Unusual LTR Retrotransposon Family in Plants
Source: PLoS One. 2012 Oct 31;7(10):e48595. doi: 10.1371/journal.pone.0048595 (PMC3485330; doi:10.1371/journal.pone.0048595)
Supplement: Table S4 — Regulatory signals in the LTR region of Retrosat2 . (DOC) [file pone.0048595.s010.doc]

Table S4. Regulatory signals in LTR region of Retrosat2

| Names | Organism | Position | Sequences | Functions |
| --- | --- | --- | --- | --- |
| A-box | Petroselinum crispum | 447+6 | CCGTCC | cis-acting regulatory element |
| A-box | Petroselinum crispum | 1041+6 | CCGTCC | cis-acting regulatory element |
| A-box | Petroselinum crispum | 1081+6 | CCGTCC | cis-acting regulatory element |
| A-box | Petroselinum crispum | 1105+6 | CCGTCC | cis-acting regulatory element |
| A-box | Petroselinum crispum | 1380+6 | CCGTCC | cis-acting regulatory element |
| C-repeat/DRE | Arabidopsis thaliana | 1227+8 | TGGCCGAC | regulatory element involved in cold- and dehydration-responsiveness |
| CAAT-box | Glycine max | 209+5 | CAATT | common cis-acting element in promoter and enhancer regions |
| CAAT-box | Glycine max | 229+5 | CAATT | common cis-acting element in promoter and enhancer regions |
| CAAT-box | Arabidopsis thaliana | 208+5 | CCAAT | common cis-acting element in promoter and enhancer regions |
| CAT-box | Arabidopsis thaliana | 1133+6 | GCCACT | cis-acting regulatory element related to meristem expression |
| CCGTCC-box | Arabidopsis thaliana | 447+6 | CCGTCC | cis-acting regulatory element related to meristem specific activation |
| CCGTCC-box | Arabidopsis thaliana | 1041+6 | CCGTCC | cis-acting regulatory element related to meristem specific activation |
| CCGTCC-box | Arabidopsis thaliana | 1081+6 | CCGTCC | cis-acting regulatory element related to meristem specific activation |
| CCGTCC-box | Arabidopsis thaliana | 1105+6 | CCGTCC | cis-acting regulatory element related to meristem specific activation |
| CCGTCC-box | Arabidopsis thaliana | 1380+6 | CCGTCC | cis-acting regulatory element related to meristem specific activation |
| G-box | Zea mays | 525+6 | CACGTC | cis-acting regulatory element involved in light responsiveness |
| G-box | Zea mays | 654+6 | CACGTC | cis-acting regulatory element involved in light responsiveness |
| G-box | Zea mays | 686+6 | CACGTC | cis-acting regulatory element involved in light responsiveness |
| GC-motif | Zea mays | 516+6 | CCCCCG | enhancer-like element involved in anoxic specific inducibility |
| GC-motif | Zea mays | 1506+7 | GCCCCGG | enhancer-like element involved in anoxic specific inducibility |
| GC-motif | Zea mays | 1543+6 | CCCCCG | enhancer-like element involved in anoxic specific inducibility |
| GC-motif | Zea mays | 1593+6 | CCCCCG | enhancer-like element involved in anoxic specific inducibility |
| GC-motif | Zea mays | 1649+6 | CCCCCG | enhancer-like element involved in anoxic specific inducibility |
| GC-motif | Zea mays | 3005+6 | CCCCCG | enhancer-like element involved in anoxic specific inducibility |
| GC-motif | Oryza sativa | 1630+8 | GCCGCGCG | ? |
| GC-motif | Oryza sativa | 722+9 | CGGCGCCCT | ? |
| GT1-motif | Arabidopsis thaliana | 2248+6 | GGTTAA | light responsive element |
| Skn-1_motif | Oryza sativa | 1959+5 | GTCAT | cis-acting regulatory element required for endosperm expression |
| Sp1 | Oryza sativa | 2716+6 | GGGCGG | light responsive element |
| ARE | Zea mays | 2220+6 | TGGTTT | cis-acting regulatory element essential for the anaerobic induction |
| ARE | Zea mays | 2285+6 | TGGTTT | cis-acting regulatory element essential for the anaerobic induction |
| Box I | Pisum sativum | 2015+7 | TTTCAAA | light responsive element |
| Box II | Petroselinum crispum | 1516+9 | CCACGTGGC | part of a light responsive element |
| C-box | Arabidopsis thaliana | 1656+10 | CTGACGTCAG | cis-acting regulatory element involved in light responsiveness |
| CCAAT-box | Hordeum vulgare | 2375+6 | CAACGG | MYBHv1 binding site |
| CGTCA-motif | Hordeum vulgare | 1660+5 | CGTCA | cis-acting regulatory element involved in the MeJA-responsiveness |
| GCN4_motif | Oryza sativa | 2361+7 | CAAGCCA | cis-regulatory element involved in endosperm expression |
| GT1-motif | Arabidopsis thaliana | 2246+6 | GGTTAA | light responsive element |
| I-box | Solanum tuberosum | 2211+9 | TGATAATGT | part of a light responsive element |
| MBS | Zea mays | 2719+6 | CGGTCA | MYB Binding Site |
| TGACG-motif | Hordeum vulgare | 1605+5 | TGACG | cis-acting regulatory element involved in the MeJA-responsiveness |
| TGACG-motif | Hordeum vulgare | 1657+5 | TGACG | cis-acting regulatory element involved in the MeJA-responsiveness |
| as1 | Arabidopsis thaliana | 1657+8 | TGACGTCA | cis-acting regulatory element involved in the root-specific expression |
| chs-CMA2a | Petroselinum crispum | 1969+8 | TCACTTGA | part of a light responsive element |
| ACE | Petroselinum hortense | 3183+8 | ACGTGGA | cis-acting element involved in light responsiveness |
