# Supplementary material for: Functional and Structural Divergence of an Unusual LTR Retrotransposon Family in Plants
Source: PLoS One. 2012 Oct 31;7(10):e48595. doi: 10.1371/journal.pone.0048595 (PMC3485330; doi:10.1371/journal.pone.0048595)
Supplement: Table S5 — Structural similarity among selected ORF0 protein members. (DOC) [file pone.0048595.s011.doc]

Table S5. Structural similarity among selected ORF0 protein members.

| RMSD (Å) | Minuta | Punctata | Rufi | RetroSat2 | RIRE3 | RIRE8 | FRetrosat3 | Sat2-off | ZmSat2 | SorSat2 | BraSat2 |
| --- | --- | --- | --- | --- | --- | --- | --- | --- | --- | --- | --- |
| Minuta | 0 | **3.991** | **6.601** | 16.959 | 15.708 | 12.884 | 17.883 | 17.039 | 17.292 | 16.419 | **9.216** |
|  | Punctata | 0 | **6.213** | 14.853 | 11.840 | 14.361 | 14.482 | 16.943 | 19.018 | 21.785 | 12.745 |
|  |  | Rufi | 0 | 16.841 | 16.106 | 14.771 | 18.412 | 17.612 | 19.533 | 14.575 | 20.212 |
|  |  |  | RetroSat2 | 0 | 12.748 | 16.586 | 12.088 | **5.693** | 21.421 | 11.149 | 20.442 |
|  |  |  |  | RIRE3 | 0 | 16.505 | **8.508** | 11.452 | 20.044 | 16.743 | 21.523 |
|  |  |  |  |  | RIRE8 | 0 | 18.239 | 16.216 | 20.152 | **9.970** | 22.128 |
|  |  |  |  |  |  | FRetrosat3 | 0 | 12.885 | 20.250 | 14.012 | 24.413 |
|  |  |  |  |  |  |  | Sat2-off | 0 | 20.388 | 16.354 | 20.283 |
|  |  |  |  |  |  |  |  | ZmSat2 | 0 | 22.568 | 17.610 |
|  |  |  |  |  |  |  |  |  | SorSat2 | 0 | 20.677 |
|  |  |  |  |  |  |  |  |  |  | BraSat2 | 0 |

Note: General structure (cartoon diagram) of the best theoretical model were superimposed by carbon a, obtaining the RMSD between each pair of structures. The structures with smaller deviations were highlighted in bold.
