# Supplementary material for: Functional and Structural Divergence of an Unusual LTR Retrotransposon Family in Plants
Source: PLoS One. 2012 Oct 31;7(10):e48595. doi: 10.1371/journal.pone.0048595 (PMC3485330; doi:10.1371/journal.pone.0048595)
Supplement: Table S6 — Functional characteristics of the ORF0 protein members based on structural features. (DOC) [file pone.0048595.s012.doc]

Table S6. Functional characteristics of the ORF0 protein members based on structural features.

| **Gene name** | **Gene ontology accession number** | **Molecular Function** | **Gene ontology accession number** | **Biological prococces** | **International E.C. Number** | **Proteins with highly similar structure** |
| --- | --- | --- | --- | --- | --- | --- |
| **Minuta** | 0004091  0004806 | Carboxylic ester hydrolase (carboxyesterase) activity  Triacylglycerol ester hydrolase activity | 0009056 0003824  0016787  0016042 | Catalytic process  Hydrolase activity  Lipid catabolic processes | 3.1.1.3.  Triacylglycerol lipase | 1tiaA  3g7nB |
| **Punctata** | 0004091  0004806 | Carboxylic ester hydrolase activity, carboxyesterase activity  Triacylglycerol ester hydrolase activity | 0009056 0003824  0016787  0016042 | Catalytic process  Hydrolase activity.  Lipid catabolic processes | 3.1.1.3.  Triacylglycerol lipase | 1tiaA  3g7nB |
| **Rufi** | 0004091  0004806 | Carboxylic ester hydrolase activity, carboxyesterase activity  Triacylglycerol ester hydrolase activity | 0009056 0003824  0016787  0016042 | Catalytic process  Hydrolase activity.  Lipid catabolic processes | 3.1.1.3.  Triacylglycerol lipase | 1tiaA  3g7nB |
| **RIRE3** | 0003943  0043169 | N-acetylgalacto/gluco-samine-4-sulfatase  Cation-binding | 0009056 0003824  0016787  0008484  0005509  0007033  0007040  0007041  0005976 | Catalytic proccess  Hydrolase activity  Sulfuric ester hydrolase activity  Ion storage activity  Vacuole (Lysosome) organization, biogenesis and transport  Polysaccharide (glycosamino-glycan) metabolic process | 3.1.6.12  Arylsulfatase activity | [1fsuA](http://www.rcsb.org/pdb/explore/explore.do?structureId=1fsu) |
| **RIRE8** | 0003677  0004520  0008270  0006281 | DNA-binding  Endodeoxyribonuclease (DNA nicking) activity  Zinc ion binding  DNA repair | 0009056 0003824  0016787  0004518  0006259  0043169  0006950 | Catalytic process  Hydrolase activity  Nuclease activity  DNA metabolic processes  Cation binding  Cellular response to stress | 3.1.21.2  Endonuclease IV  (Endonucleolytic cleavage to 5'-phosphooligonu-cleotide end-products) | 1xp3A |
| **FRetrosat3** | 0003943  0043169 | N-acetylgalacto/gluco-samine-4-sulfatase  Cation-binding | 0009056 0003824  0016787  0008484  0005509  0007033  0007040  0007041  0005976 | Catalytic process  Hydrolase activity  Sulfuric ester hydrolase activity  Ion storage activity  Vacuole (Lysosome) organization, biogenesis and transport  Polysaccharide (glycosaminoglycan) metabolic process | 3.1.6.12  Arylsulfatase activity | [1fsuA](http://www.rcsb.org/pdb/explore/explore.do?structureId=1fsu) |
| **Retrosat2** | 0003943  0043169 | N-acetylgalacto/gluco-samine-4-sulfatase  Cation-binding | 0009056 0003824  0016787  0008484  0005509  0007033  0007040  0007041  0005976 | Catalytic process  Hydrolase activity.  Sulfuric ester hydrolase activity  Ion storage activity  Vacuole (Lysosome) organization, biogenesis and transport  Polysaccharide (glycosamino-glycan) metabolic process | 3.1.6.12  Arylsulfatase activity | [1fsuA](http://www.rcsb.org/pdb/explore/explore.do?structureId=1fsu) |
| **Sat2-off** | 0003943  0046872 | N-acetylgalacto/gluco-samine-4-sulfatase  Metal ion-binding | 0009056 0003824  0016787  0008484  0005764  0005739  0009268  0007041 | Catalytic process  Hydrolase activity.  Sulfuric ester hydrolase activity  Lysosome  Mitochondrion  Response to pH  Lysosomal transport | 3.1.6.12  Arylsulfatase activity | 1fsuA |
| **ZMsat2** | 0016787 | Hydrolase activity | 0017000  0003824 | Antibiotic biosynthetic process  Catalytic process | 3.1-3.4.21  Class 3 Hydrolases | 3k3wB |
| **SorSat2** | 0004321  0016740  0000287 | Fatty-acyl-CoA synthase activity  Transferase activity  Magnesium ion binding | 0003824  0008610  0004316  0016491  0005737 | Catalytic activity  Lipid biosynthetic process  3-oxoacyl-[acyl-carrier-protein] reductase (NADPH) activity  Oxidoreductase activity  Cytoplasm | [2.3.1.86](http://ca.expasy.org/enzyme/2.3.1.86)  Fatty-acyl-CoA synthase  [2.3.1.41](http://ca.expasy.org/enzyme/2.3.1.41)  Beta-ketoacyl-acyl-carrier-protein synthase I | [2pffA](http://www.rcsb.org/pdb/explore/explore.do?structureId=2pff) |
| **BraSat2** | 0015169  0046872  0006810  0055085 | Glycerol-3-phosphate transmembrane transporter activity  Metal ion-binding  Transport protein activity (small molecules)  Transmembrane transport | 0003824  0008152  0006071  0015794  0005886 | Catalytic activity  Metabolic process  Glycerol metabolic process  Glycerol-3-phosphate transport  Plasma membrane | 2.A.1.4.3.  Glycerol-P:Pi antiporter | 3e0sA |

Note: In the table has been provided different parameters such as Gene Ontology (GO) accession number, molecular functions, biological processes, enzyme classification number and best templates analog to the query protein, to identify functional characteristics of selected ORF0 protein members.
